# BBSome function is required for both the morphogenesis and maintenance of the photoreceptor outer segment

**DOI:** 10.1371/journal.pgen.1007057

**Published:** 2017-10-19

**Authors:** Ying Hsu, Janelle E. Garrison, Gunhee Kim, Addison R. Schmitz, Charles C. Searby, Qihong Zhang, Poppy Datta, Darryl Y. Nishimura, Seongjin Seo, Val C. Sheffield

**Affiliations:** 1 Interdisciplinary Graduate Program in Molecular Medicine, University of Iowa, Iowa City, Iowa, United States; 2 Department of Pediatrics, University of Iowa, Iowa City, Iowa, United States; 3 Department of Ophthalmology and Visual Sciences, University of Iowa, Iowa City, Iowa, United States; Stanford University School of Medicine, UNITED STATES

## Abstract

Genetic mutations disrupting the structure and function of primary cilia cause various inherited retinal diseases in humans. Bardet-Biedl syndrome (BBS) is a genetically heterogeneous, pleiotropic ciliopathy characterized by retinal degeneration, obesity, postaxial polydactyly, intellectual disability, and genital and renal abnormalities. To gain insight into the mechanisms of retinal degeneration in BBS, we developed a congenital knockout mouse of *Bbs8*, as well as conditional mouse models in which function of the BBSome (a protein complex that mediates ciliary trafficking) can be temporally inactivated or restored. We demonstrate that BBS mutant mice have defects in retinal outer segment morphogenesis. We further demonstrate that removal of *Bbs8* in adult mice affects photoreceptor function and disrupts the structural integrity of the outer segment. Notably, using a mouse model in which a gene trap inhibiting *Bbs8* gene expression can be removed by an inducible FLP recombinase, we show that when BBS8 is restored in immature retinas with malformed outer segments, outer segment extension can resume normally and malformed outer segment discs are displaced distally by normal outer segment structures. Over time, the retinas of the rescued mice become morphologically and functionally normal, indicating that there is a window of plasticity when initial retinal outer segment morphogenesis defects can be ameliorated.

## Author summary

The BBSome is a protein complex that regulates ciliary trafficking in primary cilia, and mutations that impair BBSome function cause Bardet-Biedl Syndrome (BBS). BBS patients have retinal degeneration leading to blindness, but the disease pathophysiology has not been fully elucidated. In this study, we found that the BBSome is necessary for the structural organization of photoreceptor outer segments, and that the loss of different functional BBSome subunits causes outer segment malformation. Using a mouse model that allows the temporal inactivation of the BBSome, we inactivated BBSome function after the outer segment had formed normally. We found that the BBSome is required for both the initial formation and the continual maintenance of outer segment structures throughout life. In addition, using a mouse model that allows the temporal restoration of the BBSome, we restored BBSome function in immature photoreceptors and show that the malformed outer segment discs are displaced distally by normally formed outer segment structures. This finding indicates that when gene function is restored in immature retinas shortly after initial outer segment malformation, morphologically normal outer segments and a functionally normal retina can still result. This study has important implications for the timing of treatment of human retinal diseases.

## Introduction

Photoreceptor outer segments are a specialized cellular compartment organized into stacked membranous discs where proteins for the initiation of the phototransduction cascade are enriched. Proteins that do not have an active role in the outer segment are excluded from this compartment, and are retained in the inner segment or cell body. The outer segment is connected to the inner segment by a modified cilium known as the connecting cilium. This compartmentalized protein distribution in inner and outer segments is a feature of normal photoreceptor cells, and is crucial for mature photoreceptor function. However, maintaining this compartmentalized protein distribution is a complex task. Approximately 10% of the outer segment is renewed daily [1]. The outer segment membranous discs are added at the base, and old discs are shed at the distal tip and phagocytosed by the retinal pigment epithelium (RPE). Therefore, there is a constant flux of new materials moving from the inner segment to the outer segment through the connecting cilium, which is analogous to the transition zone structure found in primary cilia [2]. Maintaining a highly compartmentalized protein distribution between the inner and outer segments in this environment of high directional flux calls for mechanisms that either prevent unauthorized entry of non-outer segment proteins, and/or retrieve proteins that erroneously enter the outer segment by retrograde transport [3]. The BBSome, a protein complex consisting of BBS1, BBS2, BBS4, BBS5, BBS7, BBS8, BBS9, and BBS18 (also known as BBIP1) [4–13], regulates protein trafficking in the primary cilium. Recently, the BBSome was found to regulate the localization of more than a hundred proteins in photoreceptors to the inner segment, and prevent their accumulation in the outer segment. In the absence of BBSome function in *Lztfl1* (also known as *Bbs17*, a regulator of BBSome ciliary localization [14]) mutant mice, numerous proteins including the vesicle fusion protein syntaxin-3 (STX3) aberrantly accumulate in the photoreceptor outer segment [15], highlighting the critical requirement for BBS proteins in regulating outer segment protein composition and photoreceptor function.

Mutations disrupting BBSome function cause Bardet-Biedl Syndrome (BBS), an autosomal recessive disorder. BBS patients [16, 17] and mouse models [18–21] have retinal degeneration leading to blindness. Disorganized photoreceptor outer segments have been observed in 4–5 week old *Bbs2*^-/-^, *Bbs4*^-/-^, and *Bbs6*^-/-^ mice [22] and in 6 week old *Lztfl1*^*-/-*^ *(Bbs17*^*-/-*^*)* and *Bbs1*^M390R/M390R^ mutant mice [15]. However, it is unclear whether this phenotype is a consequence of photoreceptor degeneration or whether BBS proteins participate in initial outer segment morphogenesis, a process that takes place between postnatal day 9 to 25 in mice, [1] and between late-gestational to postnatal months in humans [23]. The requirement for BBS proteins in the continual maintenance and renewal of mature outer segments has also not been investigated. Due to the lack of understanding of when the BBSome is needed during eye development and maturation, and whether photoreceptors that initially developed without the BBSome can be rescued upon restoration of the BBSome, it is unclear when the optimal time window is for the prevention of retinal phenotypes in BBS. Understanding the role and temporal requirement of the BBSome in the developing and mature retina would contribute knowledge for the design and administration of interventions.

In this report, we address several key questions utilizing three mouse models of BBS8 (a BBSome component) that allow for the temporal deletion or restoration of the *Bbs8* gene. First, we monitored the initial formation of outer segments in congenital *Bbs8*^*-/-*^ mice by transmission electron microscopy (TEM) to determine whether the disc morphogenesis process is disrupted prior to notable photoreceptor cell loss. Using a mouse model that allows the inducible deletion of *Bbs8*, we studied the consequence of BBSome loss after outer segments have been normally formed, and investigated whether loss of the BBSome in mature outer segments causes a disruption in outer segment structure. We utilized a *Bbs8* mutant mouse line in which a gene trap can be removed using a tamoxifen-inducible FLP recombinase to restore the BBSome during the period of outer segment formation. We then assessed the function and morphology of the retina in these mice to determine the efficacy of rescue.

## Results

### Temporal expression of BBS proteins in the developing mouse eye

**Fig 1:**
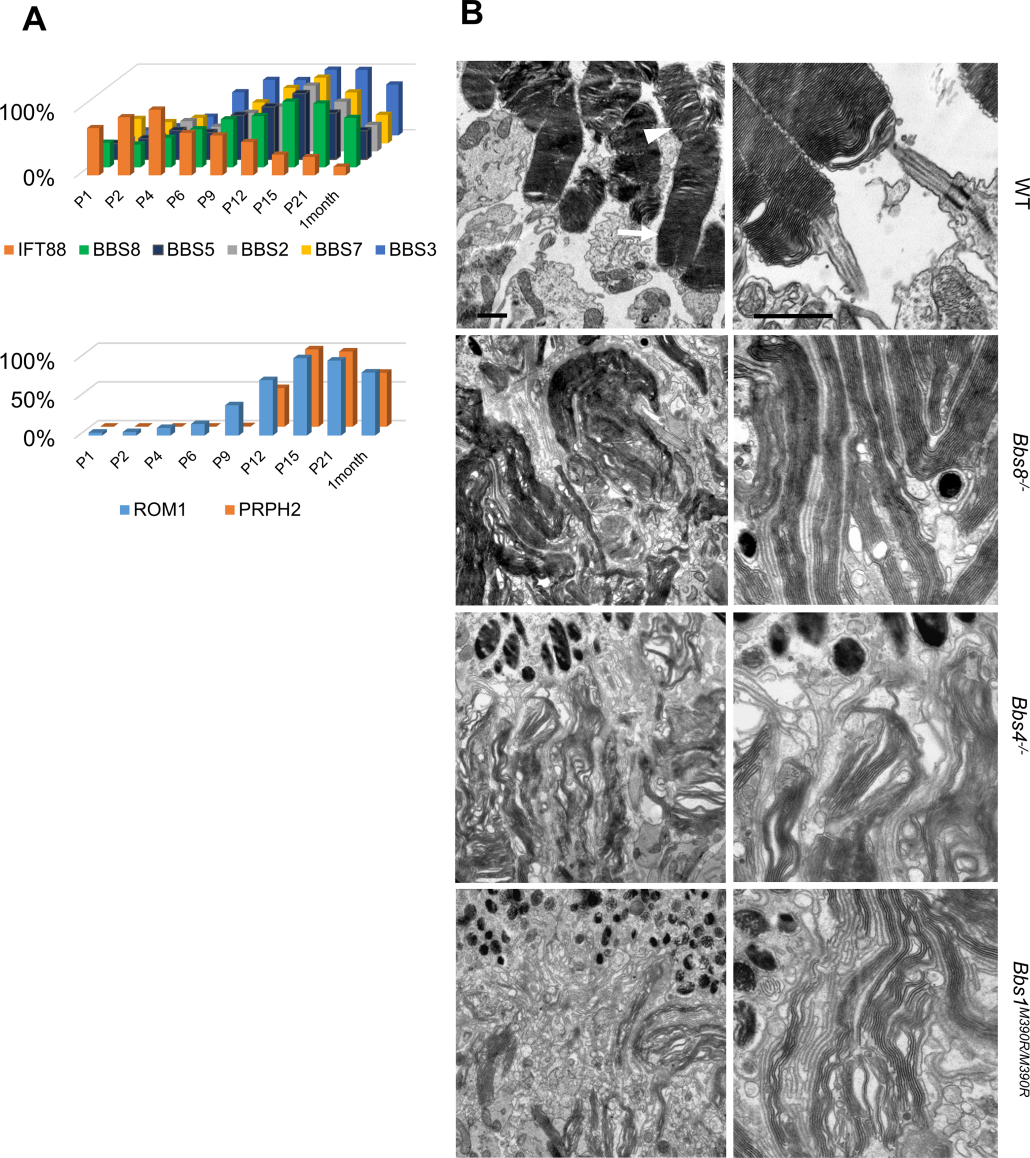
Outer segment ultrastructure in *Bbs8*^*-/-*^, *Bbs4*^*-/-*^, and *Bbs1*^*M390R/M390R*^ mice at P15. (A) Western blotting using whole eye lysates of wild type mice shows expression levels of BBS proteins, ROM1, PRHP2, and IFT88 from P1 to P30. (B) Transmission electron micrographs of outer segment ultrastructure in P15 wild type, *Bbs8*^*-/-*^, *Bbs4*^*-/-*^, and *Bbs1*^*M390R/M390R*^ mice. In these micrographs, the retinal pigment epithelium is found at the top of the images. Scale bar represents 1 μm in electron micrographs.

In order to discern when BBSome proteins are expressed in relation to the timing of photoreceptor development, we performed Western blot analyses of BBS proteins on whole eye lysates of wild type mice at postnatal (P) day 1, 2, 4, 6, 9, 12, 15, 21, and at 1 month. In mice, photoreceptors become post-mitotic by P3-5 [24], and undergo terminal differentiation. After the polarization of their apico-basal domains, the connecting cilium emerges from the apical surface around P3-P6 [25–27] and extends towards the RPE. Subsequently, nascent discs emerge from the connecting cilium around P9, are organized into columnar structures that undergo rapid extension between P12-P21 [28], and mature outer segment morphology containing compacted and well-organized discs is observed between P20-P25 [1, 29]. Of note, the expression of BBS proteins including BBS2, BBS3, BBS5, BBS7, and BBS8 demonstrates a parallel increase in expression, peaking simultaneously at P15 coincident with the height of outer segment morphogenesis, followed by a decrease thereafter (Fig 1A, S1 Fig). At one month of age, BBS protein levels have dropped to 50–75% of their peak levels at P15. The postnatal expression pattern of BBS proteins resembles those of proteins required for outer segment formation, such as peripherin-2 (PRPH2) and rod outer segment membrane protein-1 (ROM1). In contrast, the expression pattern of intraflagellar transport protein 88 (IFT88) and IFT57, members of IFT complex B [30], is distinct from that of BBS proteins, and their expression reaches peak levels between P4-P9. The temporal expression pattern of IFT88 and IFT57 instead resembles proteins related to ciliogenesis such as microtubule-actin crosslinking factor 1 (MACF1) (Fig 1A, S1 Fig). The temporal expression pattern of BBS proteins correlates with those of proteins participating in outer segment formation, and is distinct from those of proteins required for ciliogenesis.

### Mice lacking BBSome function have defects in outer segment morphogenesis

*Bbs8*^*-/-*^ mice were generated as described in the methodology (S2 Fig) and display BBS phenotypes. Confirming prior findings that the knockdown of BBS8 causes partial disassembly of the BBSome [14], there is no intact BBSome in the eyes and testis of *Bbs8*^*-/-*^ mice as shown by sucrose gradient fractionation (S3 Fig). In wild type mice, BBSome components BBS1, BBS2, BBS4, BBS5, BBS7, and BBS9 are mainly found in fraction 13, whereas these components are found in lower-molecular weight fractions in the eyes and testes of *Bbs8*^*-/-*^ mice. The shift of these components towards lower molecular weight fractions indicates the loss of the intact BBSome in *Bbs8*^*-/-*^ mice.

**Fig 2:**
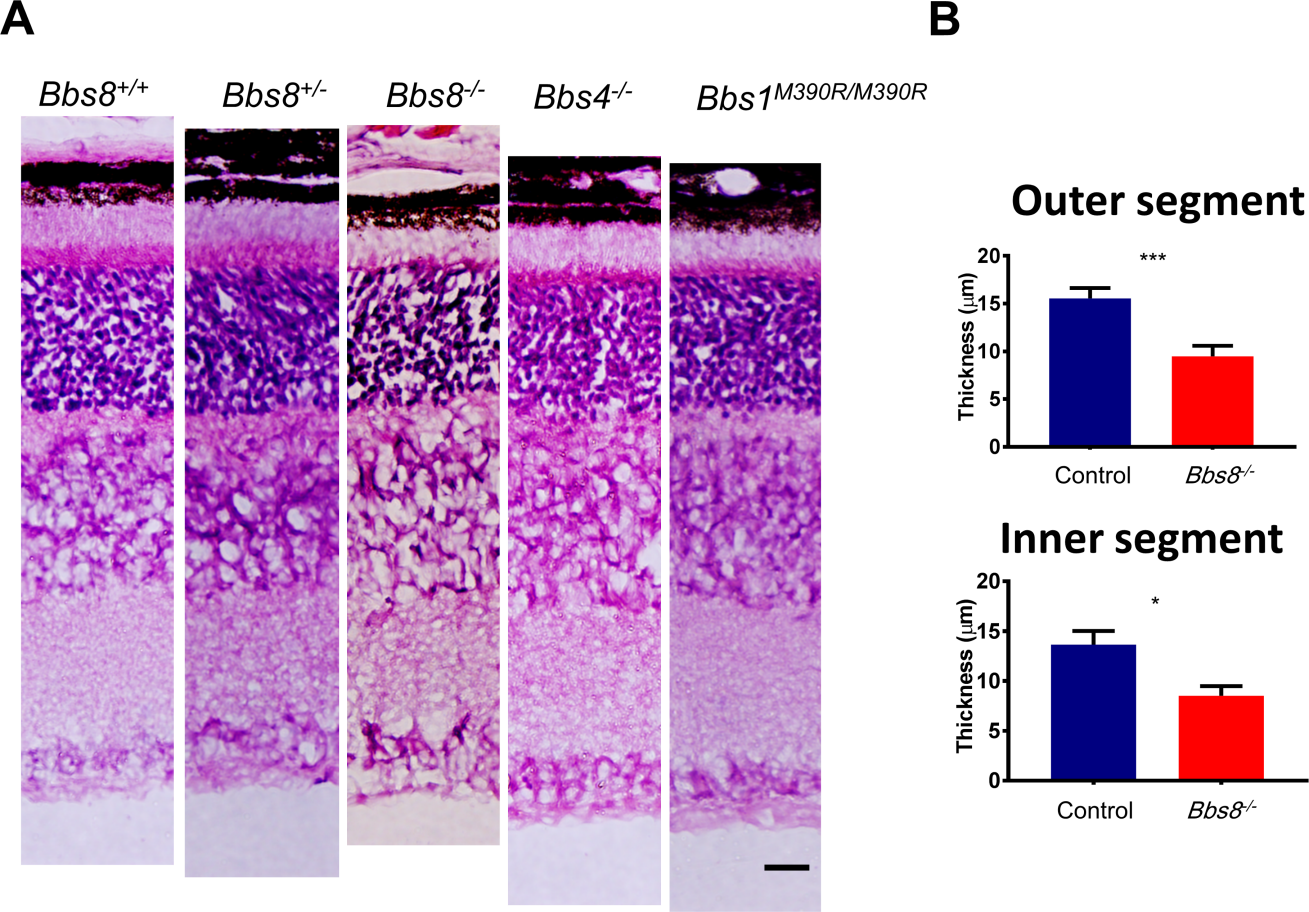
Histology of *Bbs8*^*-/-*^, *Bbs4*^*-/-*^, *and Bbs1*^*M390R/M390R*^ retinas at P15. (A) H and E histology of *Bbs8*^*-/-*^, *Bbs4*^*-/-*^, *and Bbs1*^*M390R/M390R*^ retinas at P15 compared to wild type and heterozygous littermates. Scale bar represents 20 μm. (B) Quantification of inner and outer segment lengths in *Bbs8*^*-/-*^ retinas and control littermates shows shortened inner and outer segments. Statistical significance using two-tailed *t*-test is shown where * denotes p<0.05, ** denotes p < 0.01, and *** denotes p < 0.005.

To determine whether BBSome function is specifically required for outer segment formation, the outer segment ultrastructure of *Bbs8*^*-/-*^ retinas was examined by transmission electron microscopy (TEM). TEM analyses at P15 reveal that the outer segments of *Bbs8*^*-/-*^ mice are highly disorganized, characterized by elongated discs that are vertically oriented, whereas the morphology of developing outer segments in wild type or heterozygous control littermates resemble mature outer segments with tightly stacked discs (Fig 1B). It is worth noting that while the proximal portions of outer segments are compact and tightly-stacked in wild type mice at P15 (Fig 1B, arrows), the disc lamellae in the distal portions are sometimes dilated and loosely stacked (Fig 1B, arrowheads) due to the fact that the outer segment has not fully matured yet at this age [1, 28]. Therefore, in wild type mice, these dilated and disorganized discs at the distal portions of the outer segments likely represent the immature outer segments formed a few days prior to this time point when the photoreceptors have not yet acquired the ability to organize mature outer segment structure. In order to examine whether outer segment malformation is a phenotype specific to *Bbs8*^*-/-*^ mice or a phenotype shared by mutant mice of BBSome components, we examined the outer segment ultrastructure of *Bbs1*^*M390R/M390R*^ mice [20], which model the most common human mutation in BBS, and *Bbs4*^*-/-*^ mice [19]. Even though BBS1 and BBS4 are peripheral subunits of the BBSome and are added later to the BBSome than other subunits [9], BBS1, BBS4 and BBS8 are all members of the BBSome and indispensable for BBSome function [14, 19, 20]. TEM micrographs of outer segments of *Bbs1*^*M390R/M390R*^ mice and *Bbs4*^*-/-*^ mice also show outer segment morphogenesis defects at P15 (Fig 1B). At this age, we also observed shortened inner and outer segments in retinal histology of *Bbs8*^*-/-*^, *Bbs4*^*-/-*^, and *Bbs1*^*M390R/M390R*^ mice (Fig 2) at a time point when there is only a mild reduction in the thickness of the outer nuclear layer (S4 Fig). We also confirmed that heterozygous littermates are comparable to wild type littermates (Fig 2), consistent with the fact that BBS is a recessive disorder. Both heterozygous and homozygous wild type littermates were used as controls for the study. Therefore, *Bbs8*^*-/-*^ mice have an outer segment morphogenesis defect, a phenotype that is shared by mutant mice of other components of the BBSome such as *Bbs4*^*-/-*^ and *Bbs1*^*M390R/M390R*^. We conclude that BBS mutant mice examined have outer segment morphogenesis defects caused by the lack of BBSome function.

### Photoreceptor connecting cilia are elongated in *Bbs8*^*-/-*^ mice

The outer segment in photoreceptor cells is connected to the inner segment by the connecting cilium, which is equivalent to the transition zone in primary cilia [2]. Mice with retina-specific knockout of *Ift88* display abolished ciliogenesis in photoreceptors [26]. While disabling BBSome function does not abolish ciliogenesis in cell culture, it is not known whether ciliogenesis in photoreceptor cells is affected by the loss of the BBSome. Therefore, we examined whether photoreceptors in *Bbs8*^*-/-*^ mice have ciliogenesis defects by investigating polarity, ciliogenesis frequency, and connecting cilia length.

**Fig 3:**
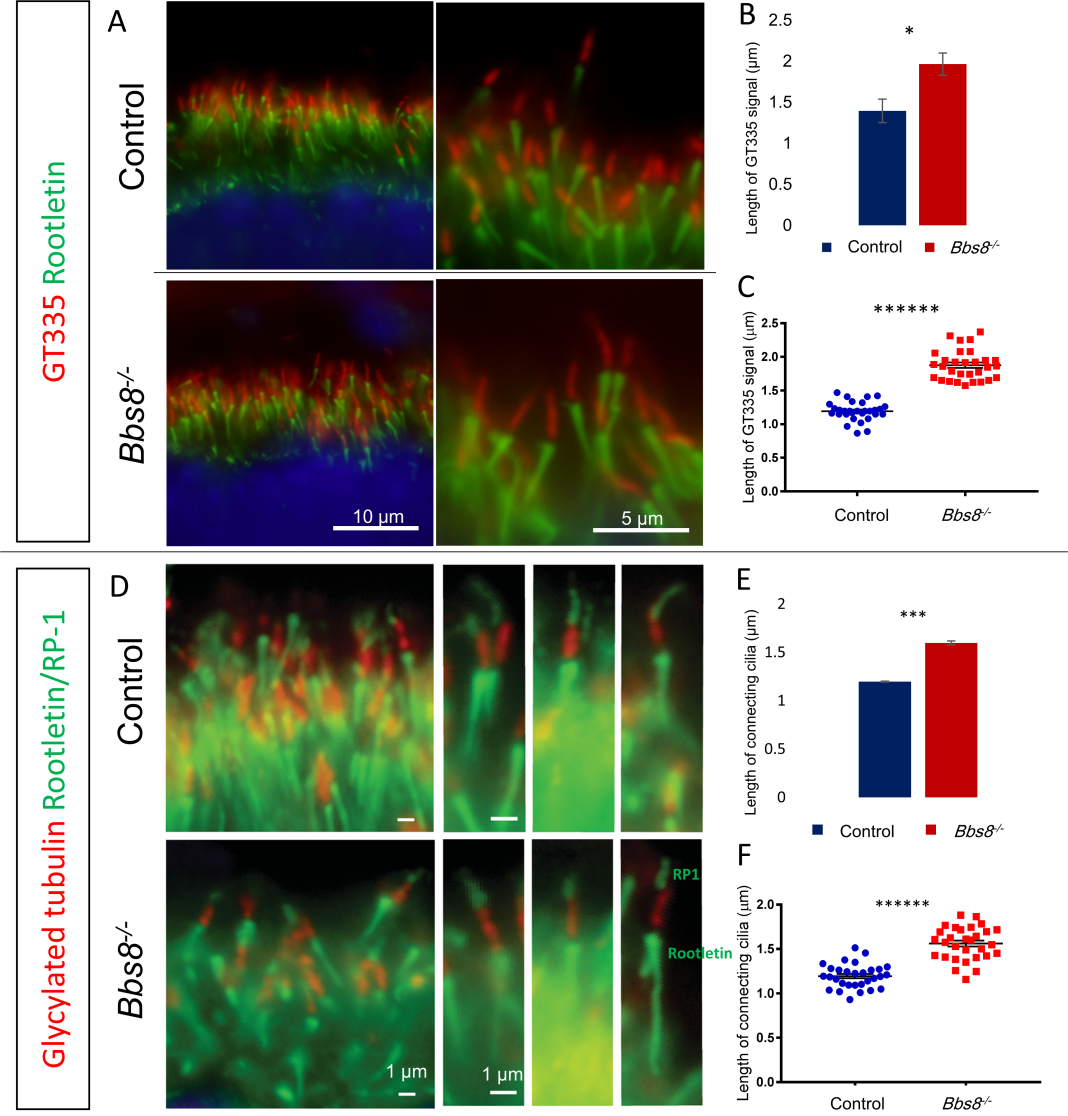
Photoreceptor connecting cilia are elongated in P15 *Bbs8*^*-/-*^ mice. (A) Elongation of GT335 signal in *Bbs8*^*-/-*^ mice compared to *Bbs8*^*+/+*^ *or Bbs8*^*+/-*^ control littermate. (B) Quantification of the length of GT335 signal in three *Bbs8*^*-/-*^ mice and their control littermates. (C) One representative experiment showing the length of GT335 signal in 30 connecting cilia of one *Bbs8*^*-/-*^ mouse and its littermate control. (D) Connecting cilia are labeled by anti-glycylated tubulin (TAP952) antibody and the connecting cilia boundaries are demarcated by rootletin (below the connecting cilia) and RP1 (above the connecting cilia). (E) Length of the glycylated tubulin (red) signal shows the elongation of the connecting cilia compartment in *Bbs8*^*-/-*^ mice. (F) One representative experiment showing length distribution of 30 connecting cilia in one *Bbs8*^*-/-*^ mouse and its littermate control. Statistical significance using two-tailed *t*-test is shown where * denotes p<0.05, ** denotes p < 0.01, *** denotes p < 0.005, **** denotes p < 0.001, ***** denotes p < 0.0005, ****** denotes p < 0.0001.

We used anti-polyglutamylation modification (GT335) and anti-glycylated tubulin (TAP952) antibodies to mark the connecting cilium. To confirm that the connecting cilia originate from the apical domain of photoreceptor cells, we co-stained the connecting cilia together with PALS1 (also known as membrane palmitoylated protein 5, MMP5), a member of the Crumbs polarity complex, which polarizes to the apical domain of photoreceptor cells during their differentiation [31]. Connecting cilia are found in the apical domain of photoreceptors in both control and *Bbs8*^*-/-*^ mice, and the PALS1 signal is localized to the apical domain and inner segment of photoreceptors in both control and *Bbs8*^*-/-*^ mice (S5 Fig). We do not observe a notable change in polarity or site of ciliogenesis due to the loss of BBS8. We also examined whether there is a change in ciliogenesis frequency. Quantification of the number of connecting cilia as marked by anti-GT335 antibody per unit length of the retina (Fig 3A) shows that photoreceptor ciliogenesis frequency is not altered in *Bbs8*^*-/-*^ mice (Control: 16.46 ± 0.27 connecting cilia; *Bbs8*^*-/-*^: 16.44 ± 0.11 connecting cilia per 10 μm length of retina; p = 0.97, S6 Fig). We then measured the length of GT335 signal (Fig 3A). In these experiments, 30 connecting cilia were measured per animal by an experimenter masked to genotype, and their values were averaged to provide a single number representing the connecting cilium length in that animal. Three animals were measured for each genotype. We noticed an unexpected elongation of the GT335 signal in *Bbs8*^*-/-*^ retinas (Control: 1.39 ± 0.14 μm, n = 3 versus *Bbs8*^*-/-*^: 1.96 ± 0.14 μm, n = 3; p < 0.05, Fig 3B). A representative experiment showing length distribution of GT335 signal in 30 connecting cilia of one *Bbs8*^*-/-*^ mouse and a littermate control is shown in Fig 3C.

Our observation of elongated GT335 signal could arise from (1) elongation of the connecting cilia compartment, or (2) expansion of the GT335 epitope beyond the connecting cilia compartment in *Bbs8*^*-/-*^ mice. These two possibilities cannot be distinguished without marking the boundary of the connecting cilia and the ciliary proper. Therefore, to distinguish which of these two possibilities is true, we demarcated the connecting cilia compartment by co-labeling a connecting cilia marker, glycylated tubulin, along with anti-RP1 antibody (which labels RP1 in the ciliary proper and represents the upper boundary of the connecting cilia) and anti-rootletin antibody (which labels the rootlets and represents the lower boundary of the connecting cilia). We observed minimal overlap of the glycylated tubulin immunofluorescence signal with either the RP1 signal or the rootletin signal in either control or *Bbs8*^*-/-*^ mice (Fig 3D). Quantification of the length of glycylated tubulin signal found between the rootletin and RP1 signals reveals that the connecting cilia in *Bbs8*^*-/-*^ mice is indeed elongated compared to their control littermates (Control: 1.1983 ± 0.0038 μm, n = 3 versus *Bbs8*^*-/-*^: 1.5985 ± 0.0211 μm, n = 3; p < 0.005, Fig 3E). A representative experiment showing length distribution of 30 connecting cilia of a *Bbs8*^*-/-*^ mouse and its control littermate is shown in Fig 3F. The elongation of the glycylated tubulin signal delimited by RP1 and rootletin supports the elongation of the connecting cilia compartment in *Bbs8*^*-/-*^ mice. Therefore, the lack of BBSome function does not abolish ciliogenesis in photoreceptors cells, but the regulation of connecting cilia length is abnormal in the absence of the BBSome.

### Syntaxin-3 mislocalizes to the outer segment in *Bbs8*^*-/-*^ retinas

**Fig 4:**
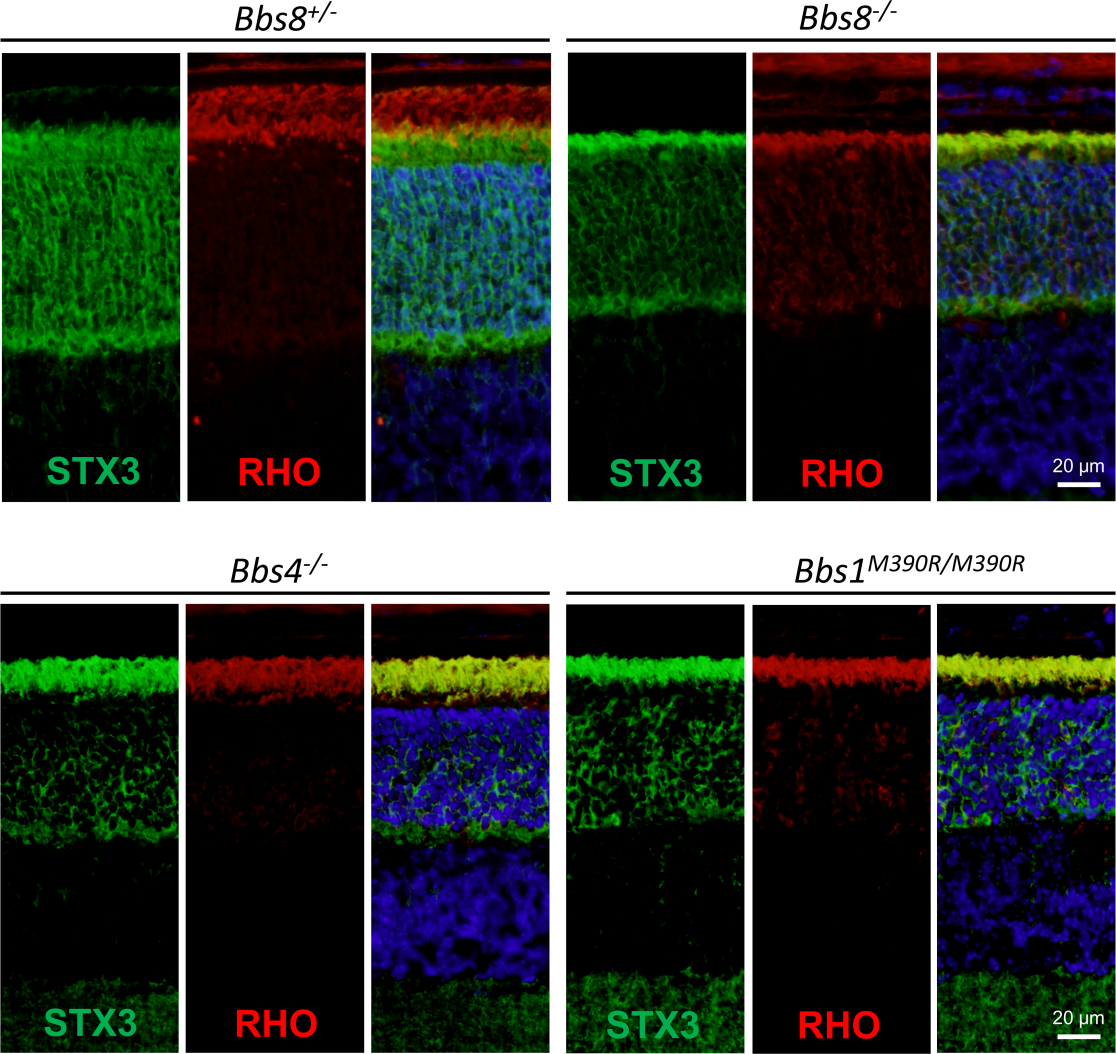
Syntaxin-3 (STX3) is mislocalized to the outer segment in P15 BBS mutant mice. In control mice, STX3 localizes to the inner segment. In *Bbs8*^*-/-*^, *Bbs4*^*-/-*^, *Bbs1*^*M390R/M390R*^ mice, STX3 mislocalizes to the outer segment, overlapping with the immunofluorescence signal of the outer segment marker, rhodopsin (RHO).

More than a hundred inner segment proteins mislocalize to the outer segment in *Lztfl1* mutant mice, including the vesicle fusion protein syntaxin-3 (STX3) [15]. Since LZTFL1 is a negative regulator of BBSome ciliary localization [14], we investigated whether loss of BBSome function due to the lack of different subunits also causes aberrant accumulation of STX3 in the photoreceptor outer segment. STX3 localizes normally to the inner segment in control mice. In *Bbs8*^*-/-*^, *Bbs4*^*-/-*^, and *Bbs1*^*M390R/M390R*^ mice, STX3 mislocalizes to the outer segment, completely overlapping with the immunofluorescence signal of rhodopsin, which marks the outer segment (Fig 4). Therefore, BBSome function is required for retaining STX3 to the inner segment of photoreceptor cells, and loss of BBSome function causes its aberrant accumulation in the outer segment.

### BBS mutant mice have low retinal function

**Fig 5:**
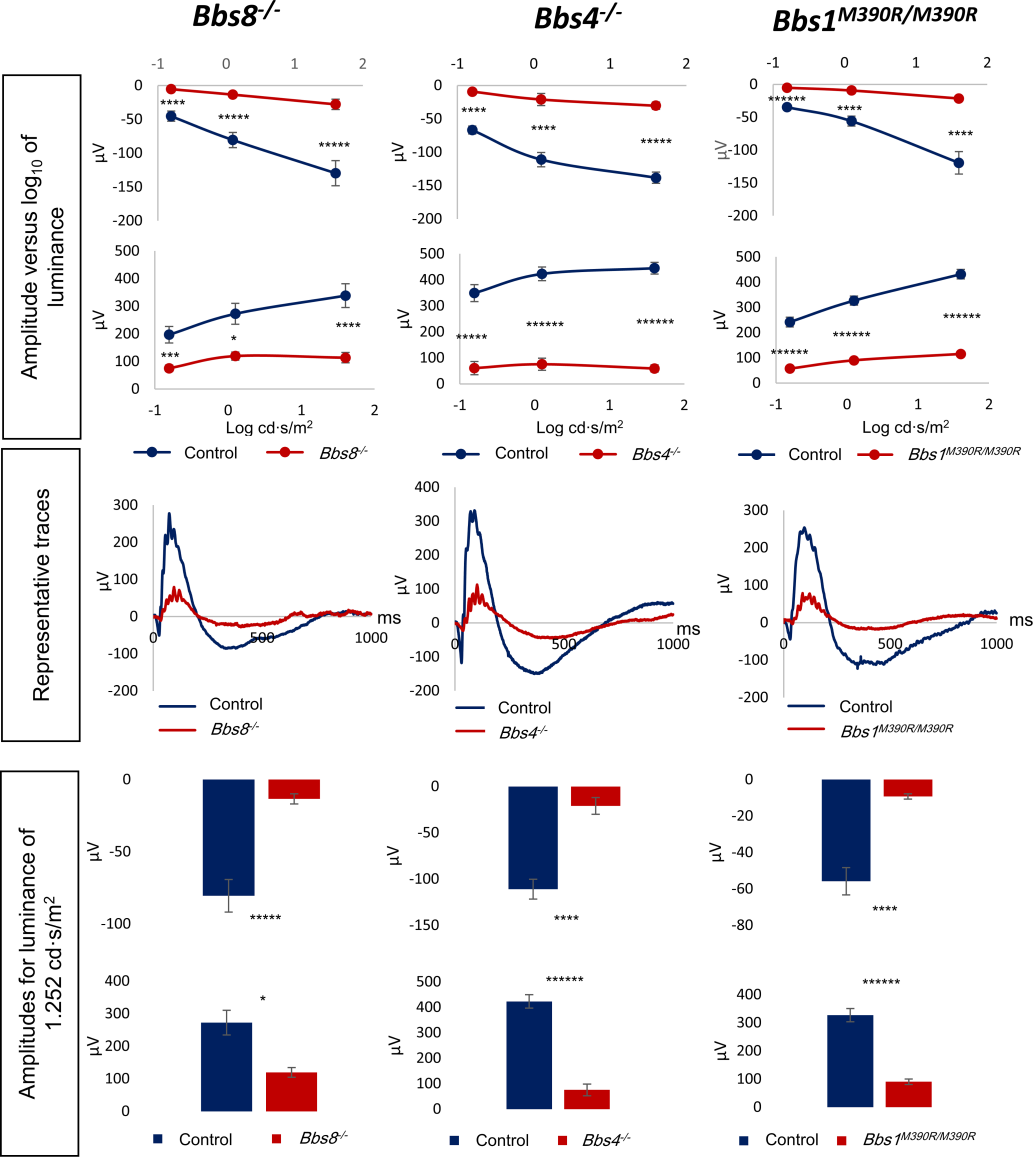
Electroretinogram (ERG) responses are diminished in *Bbs8*^*-/-*^, *Bbs4*^*-/-*^, and *Bbs1*^*M390R/M390R*^ mice. Scotopic ERG responses of P21 mutant mice are significantly reduced compared to their control wild type or heterozygous littermates. Signals of a- and b- waves (a- wave, top panel, b- wave, bottom panel), plotted against Log_10_ of luminance, are attenuated under dim (0.1564 cd·s/m^2^), intermediate (1.252 cd·s/m^2^) and bright (40 cd·s/m^2^) luminance conditions. Representative traces show the ERG response of a mutant mouse compared to its control littermate at the intermediate luminosity. Bar graphs show a- and b- wave signals (a- wave, top panel, b- wave, bottom panel) at the intermediate luminosity (1.252 cd·s/m^2^) condition.

Next, we examined the retinal function of *Bbs8*^*-/-*^, *Bbs4*^*-/-*^, and *Bbs1*^*M390R/M390R*^ mice by electroretinography (ERG) at P21, when the retina has largely matured. An ERG recording is an electrical signal elicited by retinal neurons in response to light, consisting of a negative a-wave component, and a positive b-wave component. The a-wave is induced by the hyperpolarization of the retinal photoreceptors, and the b-wave reflects the activity of the secondary neurons. Electroretinography shows that BBS mutant mice have markedly reduced a-wave and b-wave amplitudes at P21. The a- and b-wave amplitudes of all three mutants were approximately only 25%-45% of their respective controls (Fig 5). We performed a longitudinal study of ERG responses in *Bbs8*^*-/-*^ mice to evaluate whether these knockout mice reach normal a- and b- wave amplitudes at some point during retinal development and how ERG responses change during retinal degeneration. The data indicate that a- and b-wave amplitudes of *Bbs8*^*-/-*^ mice are already diminished at P15 (S7 Fig), and that these amplitudes never reach the levels of their control littermates as the retinas mature (P15, P21, 1 month, S7 Fig). The a- and b- wave amplitudes of *Bbs8*^*-/-*^ mice progressively declined thereafter due to photoreceptor degeneration and became non-recordable by 6 months of age (S7 Fig, S8 Fig). Similarly, the ERG responses of *Bbs1*^*M390R/M390R*^ and *Bbs4*^*-/-*^ mice at this time point were either residual (*Bbs1*^*M390R/M390R*^) or non-recordable (*Bbs4*^*-/-*^) (S8 Fig). At 6–7 months of age, more than 75% of the outer nuclear layer is lost in *Bbs8*^*-/-*^ mice (*Bbs8*^*-/-*^: 17.88 ± 2.53 μm, n = 4 versus wild type and heterozygous controls: 72.42 ± 4.72 μm, n = 5; p < 0.0001); the majority of photoreceptor cells are also lost in retinas of *Bbs4*^*-/-*^ and *Bbs1*^*M390R/M390R*^ mice by this time point (S9 Fig). For the models examined, null mutants of BBSome components as well as *Bbs1*^*M390R/M390R*^ possess low retinal function even before notable photoreceptor cell loss, and they have progressive retinal degeneration with the majority of photoreceptors being lost by 6–7 months of age.

### The BBSome is required for maintenance of mature photoreceptor function

We found that the lack of BBSome function disrupts the initial formation of the outer segment structure and outer segment discs are elongated and vertically oriented in mutant mice. It is not known whether the BBSome is required only during the initial formation of the outer segment or whether it is continuously needed even after outer segment maturation for its maintenance and renewal. To study the consequence of inactivating the BBSome in mature photoreceptor cells, we used a mouse model where exons 4 to 6 of the *Bbs8* gene can be deleted using a tamoxifen-inducible CRE recombinase (S2 Fig). We allowed the outer segment to develop normally in *Bbs8*^*flox/-*^*; Cre*^*+*^ mice, and then deleted the *Bbs8* gene to inactivate BBSome function at two months of age, hereafter referred to as the adult cohort. We examined retinal function, STX3 localization, and outer segment structure at several time points after BBSome inactivation. These mice were compared to a cohort in which *Bbs8* was deleted by tamoxifen injections between P9-P15 in *Bbs8*^*flox/flox*^*; Cre*^*+*^ or *Bbs8*^*flox/-*^*; Cre*^*+*^ mice, hereafter referred to as the infantile cohort. Both *Bbs8*^*flox/flox*^ and *Bbs8*^*flox/-*^ mice were used in the infantile cohort (as opposed to the adult cohort in which only *Bbs8*^*flox/-*^ mice were used) due to the apparent ease in deleting *Bbs8* in infantile mice compared to adult mice. *Cre*^*+*^ mice in either cohort that did not achieve greater than 90% excision as determined using a PCR-based assay (described in detail in the Materials and Methods section) were excluded from the study.

**Fig 6:**
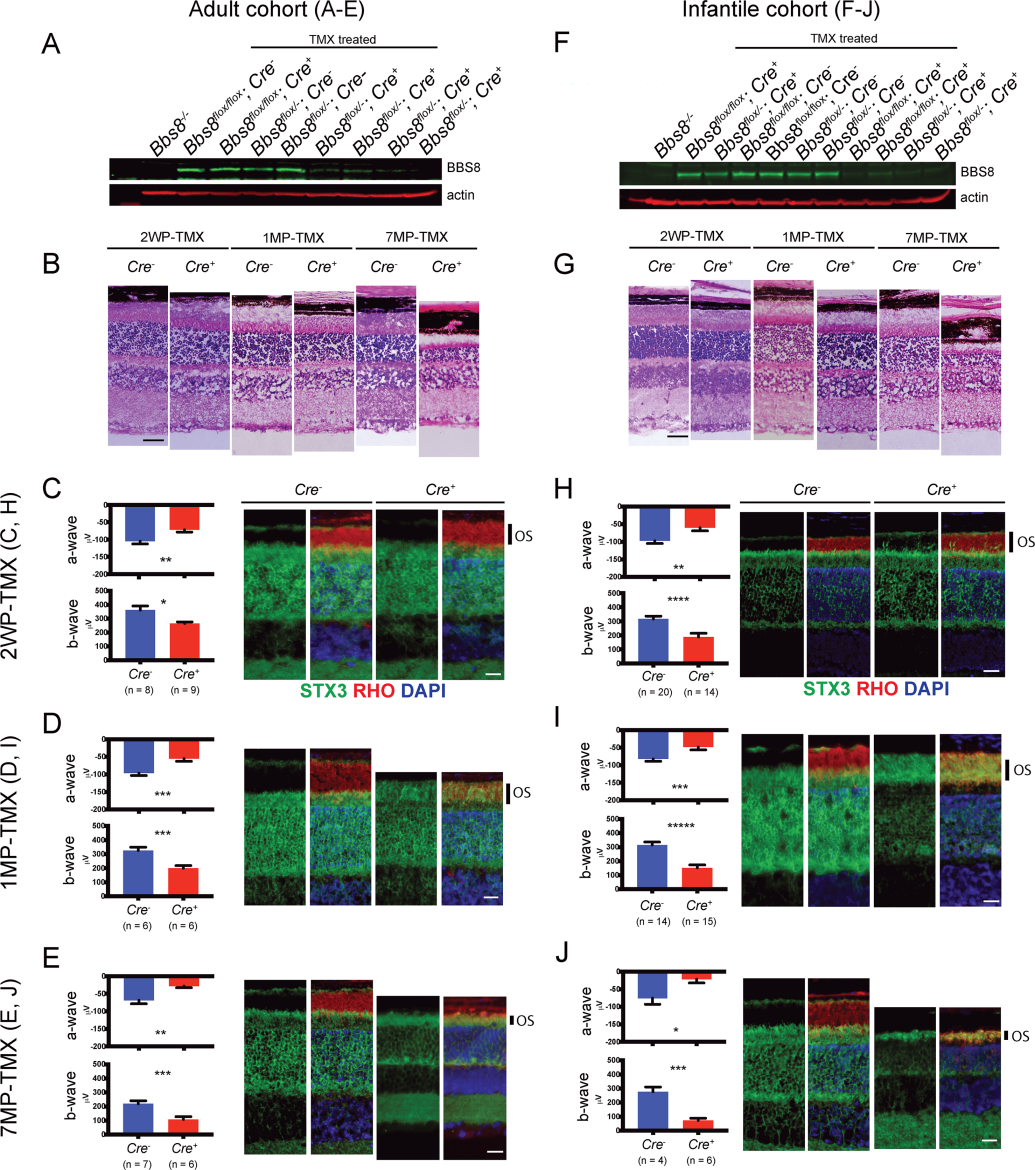
Deletion of *Bbs8* in adult and infantile mice causes retinal degeneration. (A, F) BBS8 protein levels two weeks after the end of tamoxifen injections (2 weeks post tamoxifen, 2WP-TMX) in adult (A) and infantile (F) cohorts. (B, G) Retinal histology shows progressive retinal degeneration in tamoxifen-treated mice (adult, B; infantile, G). (C, H) STX3 localization (green) and a- and b-wave amplitudes in electroretinograms (ERG) of *Cre*^*-*^ and *Cre*^*+*^ mice at 2WP-TMX (adult, C; infantile, H). The outer segment is labeled with an antibody against rhodopsin (RHO, red). (D, I) STX3 localization and ERG amplitudes at one month after the end of tamoxifen injections (1MP-TMX) (adult, D; infantile, I). (E, J) STX3 localization and ERG amplitudes at 7MP-TMX (adult, E; infantile, J). All scale bars represent 20 μm. OS: outer segment. P values for two-tailed Welsh’s test are shown: * denotes p<0.05, ** denotes p < 0.01, *** denotes p < 0.005, **** denotes p < 0.001, ***** denotes p < 0.0005.

The injection of tamoxifen in the adult and infantile cohorts resulted in comparable loss of BBS8. BBS8 protein levels were reduced by approximately 80% two weeks after the end of tamoxifen treatment in both cohorts (Fig 6A, adult; Fig 6F, infantile; quantification in S10 Fig). To ascertain that the presence or activation of CRE recombinase alone does not cause toxicity and retinal degeneration, we performed tamoxifen injections in *Bbs8*^*wt/flox*^ or *Bbs8*^*wt/wt*^*; Cre*^*+*^ mice, and confirmed that retinal degeneration was not observed in these mice (S11 Fig).

A reduction in ERG amplitudes was observed in *Bbs8*^*flox/-*^*; Cre*^*+*^ mice in the adult cohort as soon as 2 weeks after the last tamoxifen injection (2WP-TMX). Both a-wave and b-wave amplitudes were significantly reduced in these mice (2WP-TMX, Fig 6C, a-wave: -104.9 ± 7.842, n = 8, *Cre*^*-*^ versus -71.9 ± 6.7, n = 9, *Cre*^*+*^, p < 0.01; b-wave: 361.7 ± 29.41, n = 8, *Cre*^*-*^ versus 264.2 ± 11.97, n = 9, *Cre*^*+*^, p < 0.05). These reductions occurred prior to notable histological abnormalities and mislocalization of the vesicle fusion protein syntaxin-3 (STX3) (Fig 6B, histology, Fig 6C, ERG). Accumulation of STX3 in the outer segment was observed one month after the last tamoxifen injection (Fig 6D) when there was also a notable reduction in the thicknesses of the outer segment and the outer nuclear layer. The ERG responses continued to decrease with age in *Bbs8*^*flox/-*^*; Cre*^*+*^ mice, and the thickness of their outer nuclear layer was reduced by 33% seven months after the last tamoxifen injection (*Cre*^*-*^: 61.53 ± 1.31 μm, n = 4 versus *Cre*^*+*^: 41.42 ± 2.62 μm, n = 3, p < 0.01; Fig 6C, 6D and 6E, quantifications of retinal thicknesses at 4 months and 7 months after the last tamoxifen injection are shown in S12 Fig and S13 Fig). These data show that the disruption of BBSome function in adult mice causes mislocalization of STX3, reduced retinal function, and retinal degeneration.

The reduction in ERG amplitudes and mislocalization of STX3 were also observed in the infantile cohort. In the infantile cohort, both the a- and b- wave amplitudes of the *Cre*^*+*^ mice are significantly different from those of *Cre*^*-*^ mice 2 weeks after the last tamoxifen injection (2WP-TMX, Fig 6H, a-wave: -97.03 ± 7.745, n = 20, *Cre*^*-*^ versus -62.79 ± 8.762, n = 14, *Cre*^*+*^, p < 0.01; b-wave: 316.1 ± 20.1, n = 20, *Cre*^*-*^ versus 187.9 ± 26.64, n = 14, *Cre*^*+*^, p < 0.001), when STX3 still localizes to the inner segment (Fig 6H). STX3 mislocalizes to outer segment one month after the last tamoxifen injection (Fig 6I), similar to that observed in *Cre*^*+*^ mice of the adult cohort (Fig 6D). Over the course of several months, the outer nuclear layer continued to decrease in thickness in *Cre*^*+*^ mice and 59% of the outer nuclear layer was lost 7 months after the last tamoxifen injection (*Cre*^*-*^: 72.59 ± 7.23 μm, n = 3 versus *Cre*^*+*^: 29.83 ± 3.99 μm, n = 7; p < 0.05, S12 Fig and S13 Fig). In both the adult and infantile cohorts, there is progressive retinal degeneration in *Cre*^*+*^ mice as evident by reductions in thickness of the outer nuclear layers (Fig 6B, adult cohort, Fig 6G, infantile cohort). We show that the inactivation of BBSome function in mature photoreceptors causes a reduction in retinal function, mislocalization of STX3 to the outer segment, and progressive degeneration of the retina. Therefore, the BBSome is required to maintain photoreceptor function in mature eyes even after the completion of development.

**Fig 7:**
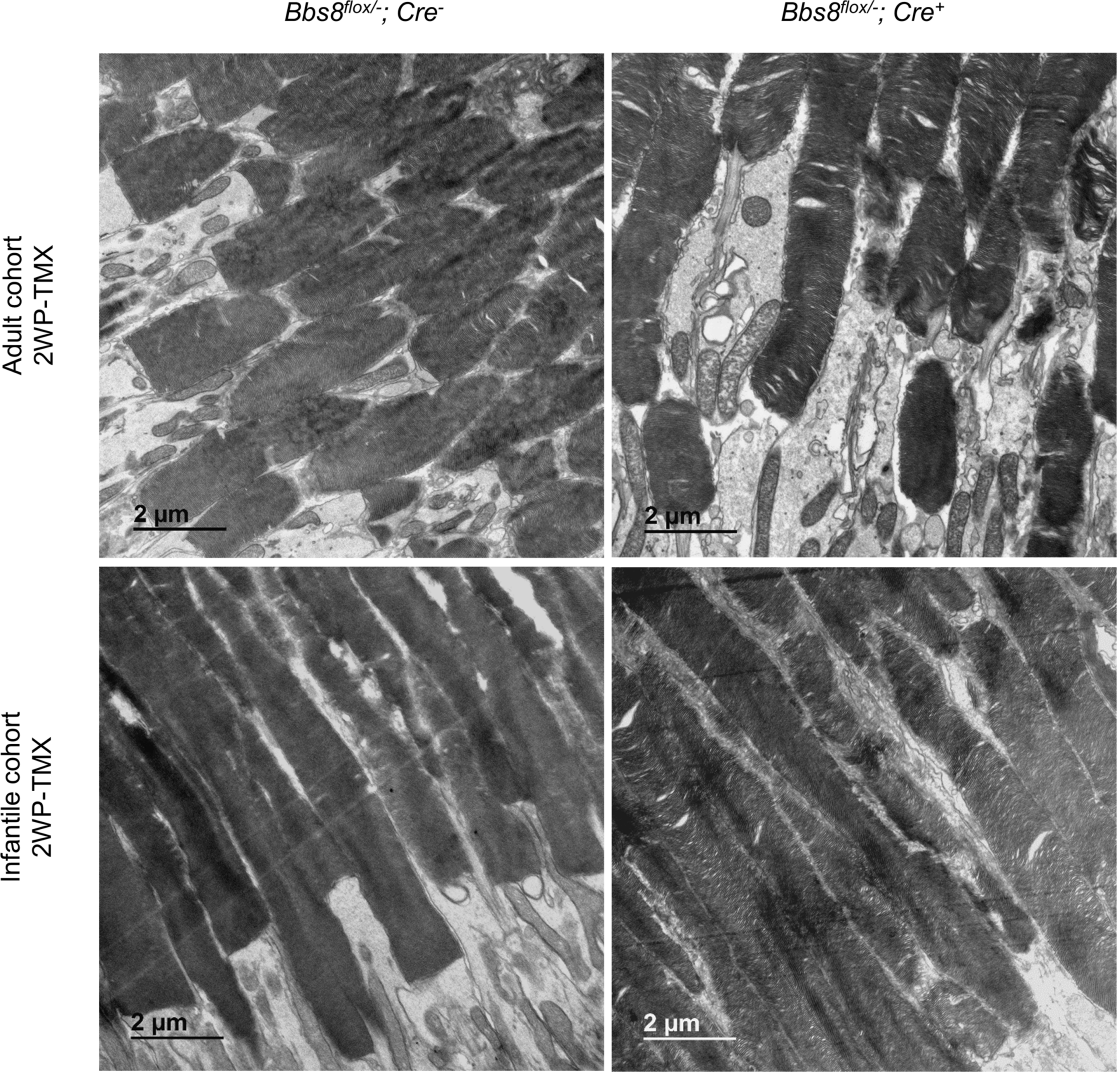
Outer segment morphology in adult and infantile cohorts 2 weeks after the last tamoxifen injection (2WP-TMX). Outer segment ultrastructure was examined by transmission electron microscopy in *Bbs8*^*flox/-*^*; Cre*^*+*^ mice compared to their injected *Cre*^*-*^ controls.

Retinal degeneration in *Cre*^*+*^ mice in the adult cohort appears to be slower than that in the infantile cohort after the deletion of *Bbs8*, despite the lack of discernable differences in the efficiency of tamoxifen induced recombination and subsequent BBS8 loss. Therefore, we performed TEM to examine whether this is due to a disruption in the initial outer segment morphogenesis in the infantile cohort, similar to that observed in *Bbs8*^*-/-*^ mice. Two weeks after the last tamoxifen injection, outer segments of *Cre*^*+*^ mice appear largely normal in both cohorts (Fig 7). This suggests that in the infantile cohort, the injections of tamoxifen at P9, P12, and P15 did not impair the initial formation of the outer segment and a sufficient reduction in BBS8 levels was only reached after the completion of outer segment morphogenesis. Injections of tamoxifen in pups younger than P9 caused a significant lethality that precluded further study. Therefore, outer segments initially formed normally in both cohorts.

**Fig 8:**
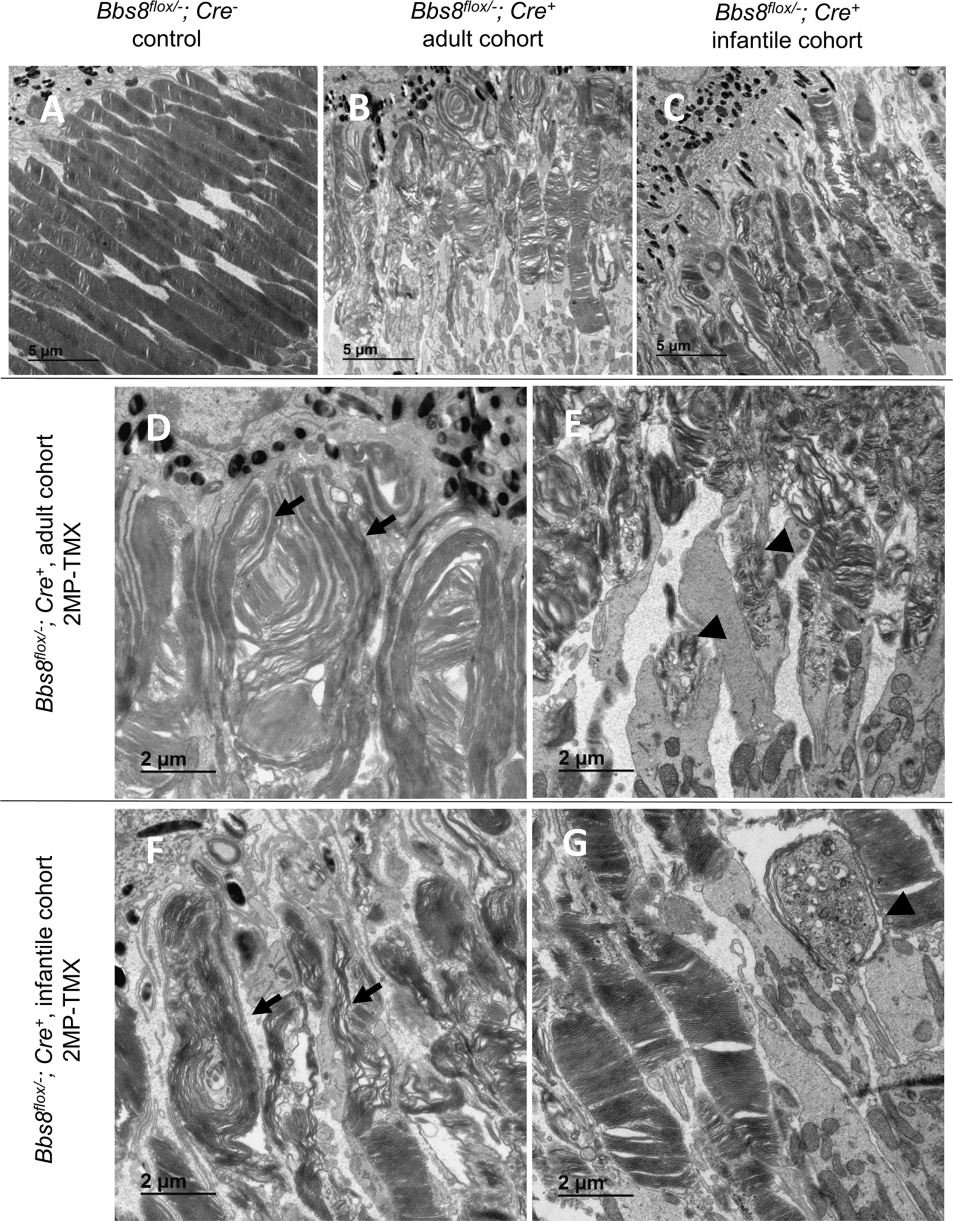
Deletion of *Bbs8* after normal development disrupts outer segment structure. TEM was performed two months after the last tamoxifen injection (two months post tamoxifen—2MP-TMX) in cohorts of both adult (B, D, E) and infantile (C, F, G) *Bbs8*^*flox/-*^ or *Bbs8*^*flox/flox*^*; Cre*^*+*^ mice. Elongated outer segments (arrows) and enlarged sacs filled with vesicles and dysmorphic disc material (arrowheads) are highlighted. Images were acquired at 1000X (A, B, C) and 2000X (D, E, F, G) magnification.

To investigate whether the BBSome continues to function in the maintenance of mature outer segment ultrastructure, we performed TEM on retinas from both the adult and infantile cohorts. Two months after tamoxifen induced deletion of *Bbs8* in adult and infantile mice, elongated and disorganized outer segments were observed (Fig 8B and 8D, arrows). The retinas of *Cre*^*+*^ mice in both cohorts contain dysmorphic outer segments characterized by elongated, vertically oriented discs (Fig 8B and 8D, adult, Fig 8C and 8F, infantile; arrows), similar to those observed in congenital knockout mice (Fig 1B). Occasionally, enlarged sacs at the end of connecting cilia filled with vesicles and dysmorphic disc materials (such as discs with small diameter and random orientation) were observed (Fig 8E, adult, and Fig 8G, infantile; arrowheads). These results show that loss of BBSome function at any time leads to impaired outer segment morphogenesis and disrupts the normal renewal of the outer segment. Therefore, BBSome function is needed not just for the initial acquisition of the outer segment structure but also for the continual maintenance and renewal of the photoreceptor outer segment throughout life.

### Functional and morphological rescue of the retina with restoration of BBS8 function

Since our data indicate that the BBSome is required for outer segment morphogenesis, we tested whether postnatal restoration of the BBSome during the period of initial outer segment formation could prevent the development of retinal phenotypes. To induce temporal restoration of the BBSome, we used a mouse model where a gene trap placed between exon 3 and 4 of the *Bbs8* gene can be excised by tamoxifen inducible FLP recombinase to restore expression of *Bbs8*. Here we denote homozygous gene-trapped animals as *Bbs8*^*gt/gt*^ to distinguish them from congenital knockout mice described previously (*Bbs8*^*-/-*^).

**Fig 9:**
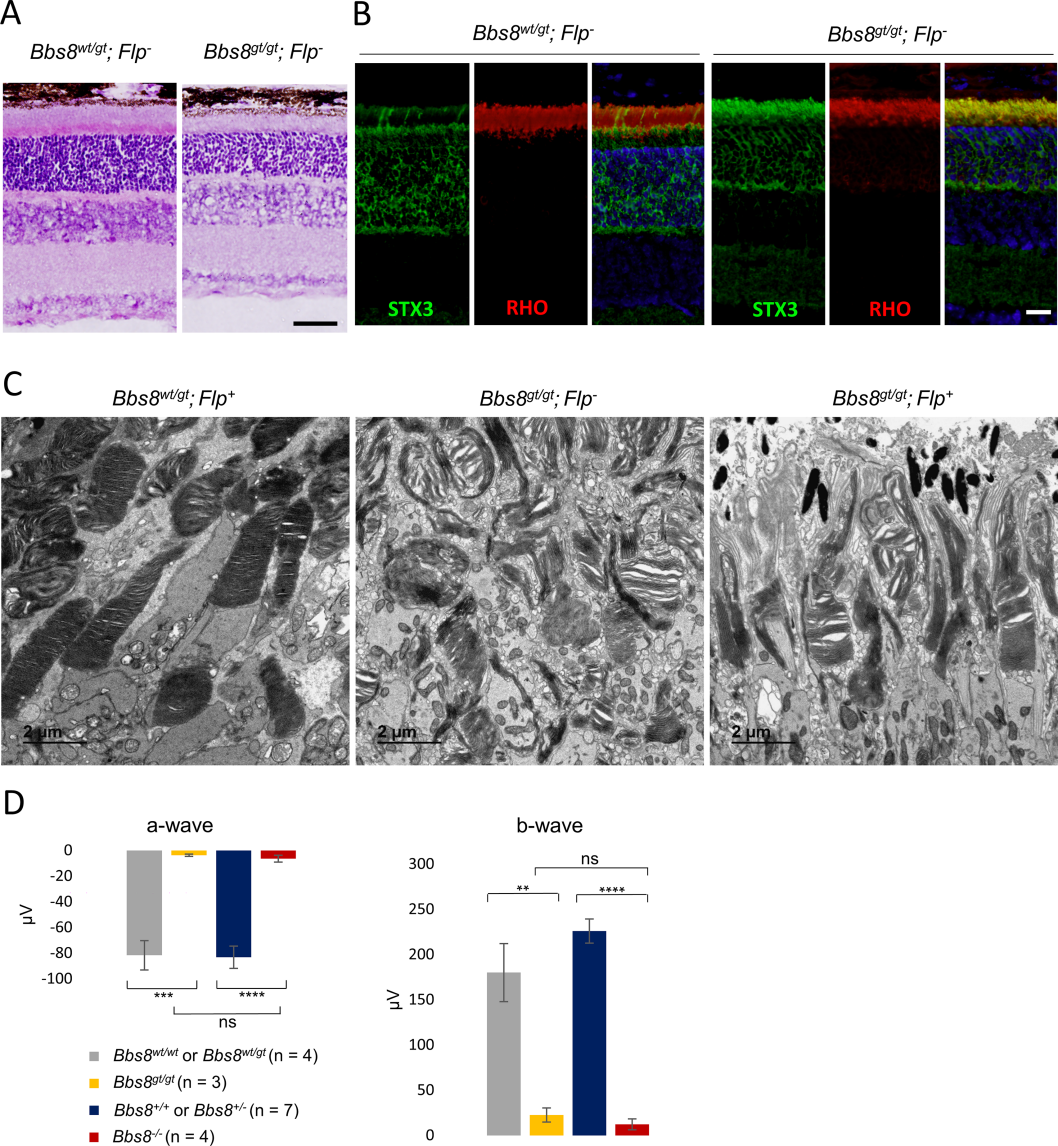
*Bbs8*^*gt/gt*^ mice recapitulate the phenotype of *Bbs8* congenital knockout (*Bbs8*^*-/-*^) mice. (A) Histology of homozygous gene-trapped mice (*Bbs8*^*gt/gt*^) mice at P15. Scale bar: 50 μm. (B) Immunofluorescence of STX3 localization in the outer segment of *Bbs8*^*gt/gt*^ mice. Scale bar: 20 μm. (C) TEM of outer segment ultra-structure of untreated *Bbs8*^*gt/gt*^*; Flp*^*-*^ and *Bbs8*^*gt/gt*^*; Flp*^*+*^ mice at P15. (D) A- and b-wave amplitudes of electroretinograms of *Bbs8*^*gt/gt*^*; Flp*^*-*^ animals and *Bbs8*^*-/-*^ animals at P15. P values for one-way ANOVA followed by post-hoc Tukey’s test are shown: * denotes p<0.05, ** denotes p < 0.01, *** denotes p < 0.005, **** denotes p < 0.001, ***** denotes p < 0.0005.

Untreated *Bbs8*^*gt/gt*^ mice recapitulate the phenotypes observed in *Bbs8*^*-/-*^ mice. Homozygous gene-trapped mice have reduced inner and outer segment lengths at P15 (Fig 9A), and STX3 mislocalizes to the outer segment (Fig 9B). In the absence of tamoxifen, both *Flp*^*-*^ and *Flp*^*+*^ *Bbs8*^*gt/gt*^ mice show disorganized outer segment morphology at P15 (Fig 9C), suggesting that homozygous gene-trapped mice lack adequate BBSome function. In addition, *Bbs8*^*gt/gt*^ mice have diminished ERG responses comparable to those of congenital knockout mice at P15 (Fig 9D).

**Fig 10:**
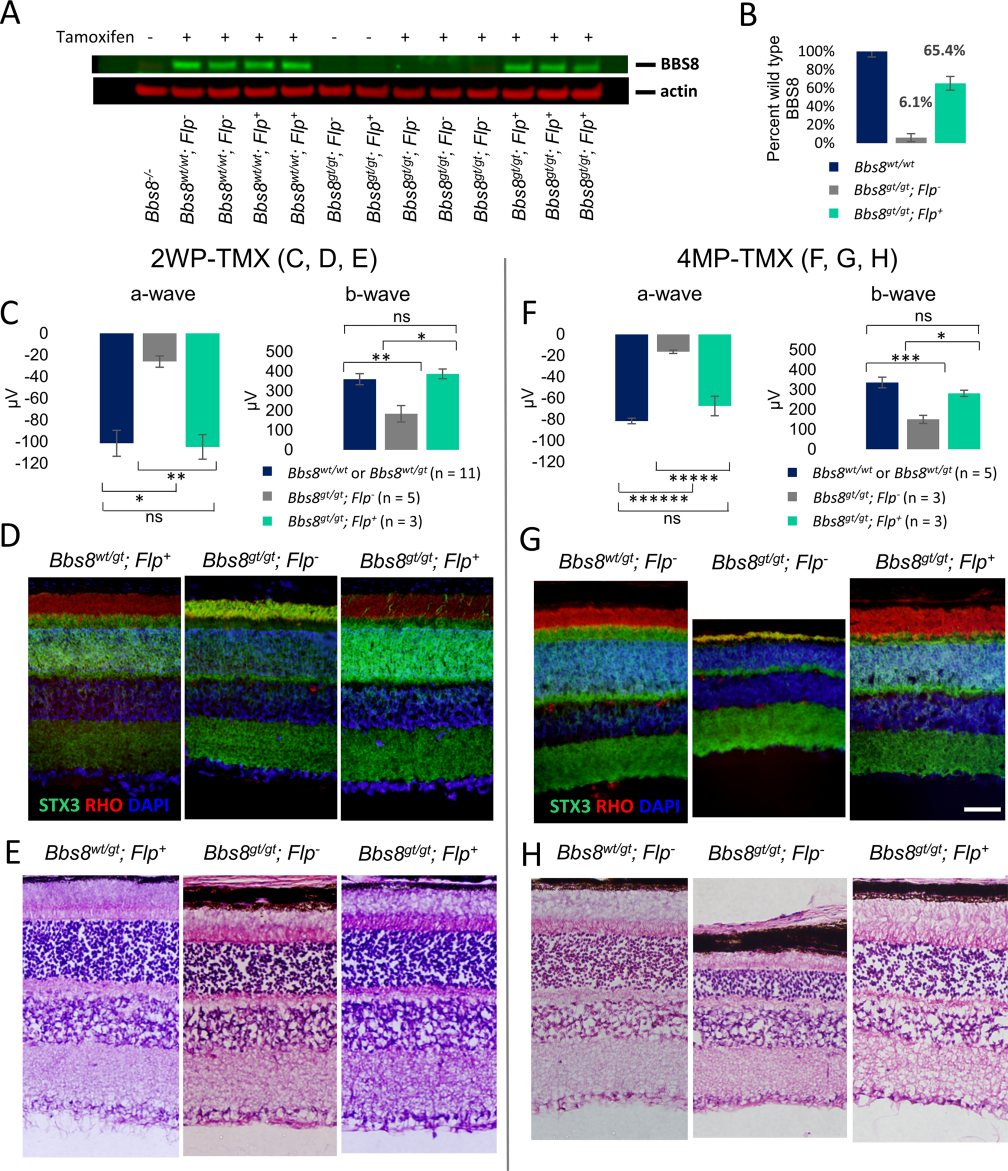
Restoration of *Bbs8* expression by tamoxifen injections between P9-P15 provides full rescue of retinal phenotypes. Mice were injected with tamoxifen between P9-P15. (A) BBS8 protein levels in one month old *Bbs8*^*gt/gt*^*; Flp*^*-*^ and *Bbs8*^*gt/gt*^*; Flp*^*+*^ mice with or without tamoxifen treatment. (B) Quantification of BBS8 band intensities in the western blot in (A) shows BBS8 proteins in rescued *Bbs8*^*gt/gt*^*; Flp*^*+*^ mice recovered to greater than 65% of BBS8 levels in wild type mice. (C) Electroretinogram (ERG) function of treated *Bbs8*^*gt/gt*^*; Flp*^*+*^ at one month of age. (D) STX3 localization in treated *Bbs8*^*gt/gt*^*; Flp*^*+*^ mice. (E) Morphological rescue of inner and outer segment thickness in *Bbs8*^*gt/gt*^*; Flp*^*+*^ mice. Perdurance of rescue is demonstrated by ERG (F), STX3 localization (G), and histology (H) at 4 months after the last tamoxifen injection (4MP-TMX). P values for one-way ANOVA followed by post-hoc Tukey’s test are shown: * denotes p<0.05, ** denotes p < 0.01, *** denotes p < 0.005, **** denotes p < 0.001, ***** denotes p < 0.0005, ****** denotes p < 0.0001. Scale bar: 50 μm.

**Fig 11:**
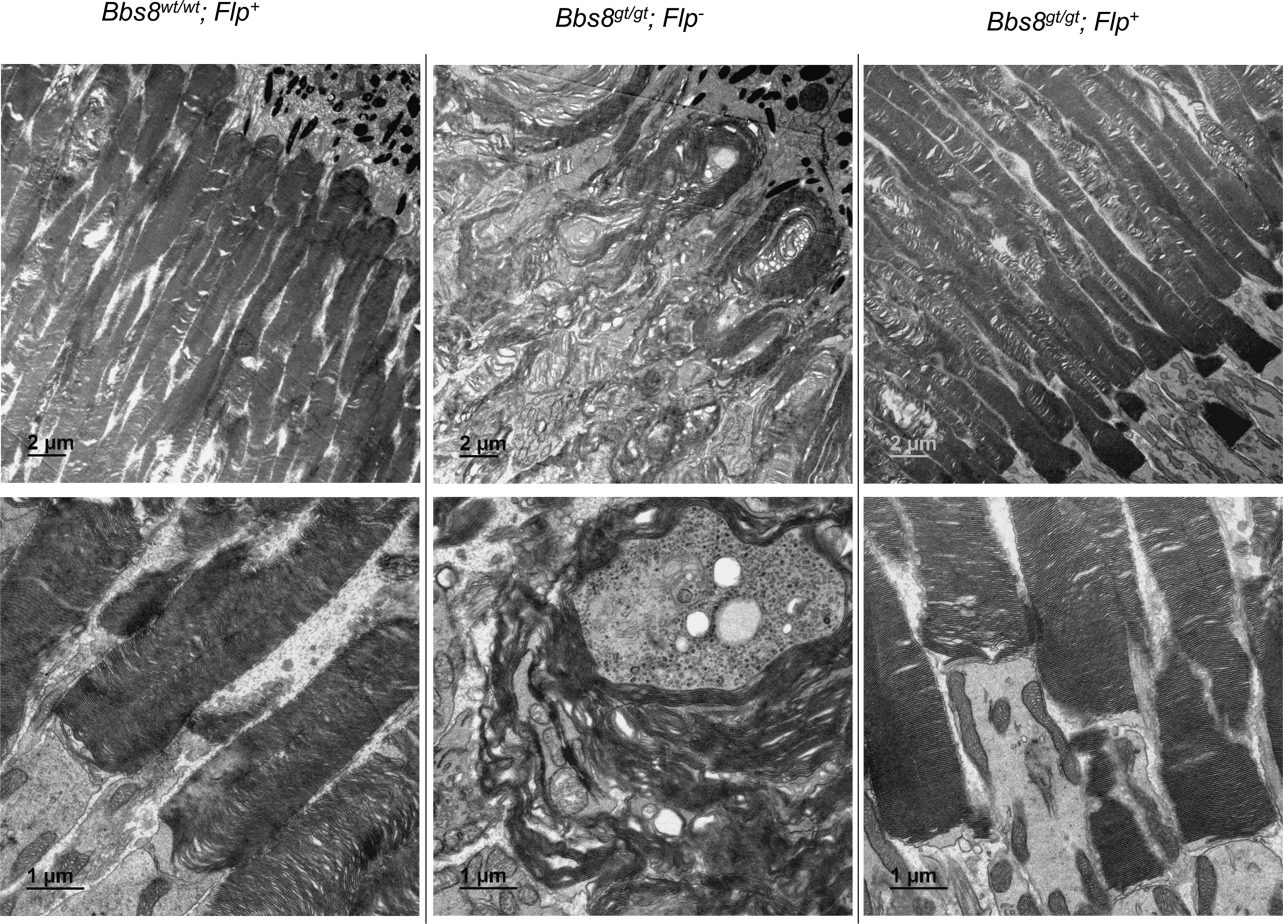
Restoration of *Bbs8* expression by tamoxifen injections between P9-P15 rescues outer segment morphology. Transmission electron microscopy was performed 4 months after the last tamoxifen injection to examine outer segment ultrastructure.

We restored *Bbs8* gene expression during the onset of outer segment formation (P9-P15) by using tamoxifen to induce FLP recombinase-mediated excision of the gene trap. BBS8 protein levels in treated and untreated mice were assessed two weeks after the final injection, which was when the animals were 1 month of age (Fig 10A). The identity of the BBS8 band was verified by its complete absence in the *Bbs8*^*-/-*^ mouse eye lysate. In uninjected *Bbs8*^*gt/gt*^*; Flp*^*-*^ and *Bbs8*^*gt/gt*^*; Flp*^*+*^ mice, the presence of BBS8 protein is extremely low. In tamoxifen-injected animals, *Bbs8*^*gt/gt*^*; Flp*^*-*^ animals still lack discernable BBS8 signal. In contrast, BBS8 protein levels in injected *Bbs8*^*gt/gt*^*; Flp*^*+*^ animals have recovered to greater than 65% of wild type levels (Fig 10B). Consistent with restored protein expression, ERG responses in rescued *Bbs8*^*gt/gt*^*; Flp*^*+*^ mice are indistinguishable from their littermate controls (a-wave: p = 0.917; b-wave: p = 0.699, ANOVA), and significantly higher than *Bbs8*^*gt/gt*^*; Flp*^*-*^ littermates (a-wave: p = 0.0062; b-wave, p = 0.0113, ANOVA; Fig 10C) at 1 month of age. In addition, STX3 is correctly localized to the inner segment in *Bbs8*^*gt/gt*^*; Flp*^*+*^ mice, similar to their control littermates, whereas STX3 mislocalizes to the outer segment in *Bbs8*^*gt/gt*^*; Flp*^*-*^ mice (Fig 10D), suggesting a preservation of compartmentalized protein distribution in the inner and outer segment. Only a few outer segments with STX3 accumulation are observed in *Bbs8*^*gt/gt*^*; Flp*^*+*^ mice. The persistence of rescue in *Bbs8*^*gt/gt*^*; Flp*^*+*^ animals was verified. ERG amplitudes (Fig 10F) and retinal histology (Fig 10H) from treated *Bbs8*^*gt/gt*^*; Flp*^*+*^ animals were indistinguishable from their control littermates 4 months after the last tamoxifen injection. STX3 localization to the inner segment is also maintained over time in these mice (Fig 10G). We performed TEM to examine outer segment ultrastructure. Transmission electron micrographs show that the outer segments of *Bbs8*^*gt/gt*^*; Flp*^*+*^ mice are indistinguishable from those of control mice, whereas the outer segment discs of *Bbs8*^*gt/gt*^*; Flp*^*-*^ mice are elongated and aberrantly oriented (Fig 11). These results show that early postnatal restoration of BBSome function can result in a functionally and morphologically normal retina.

**Fig 12:**
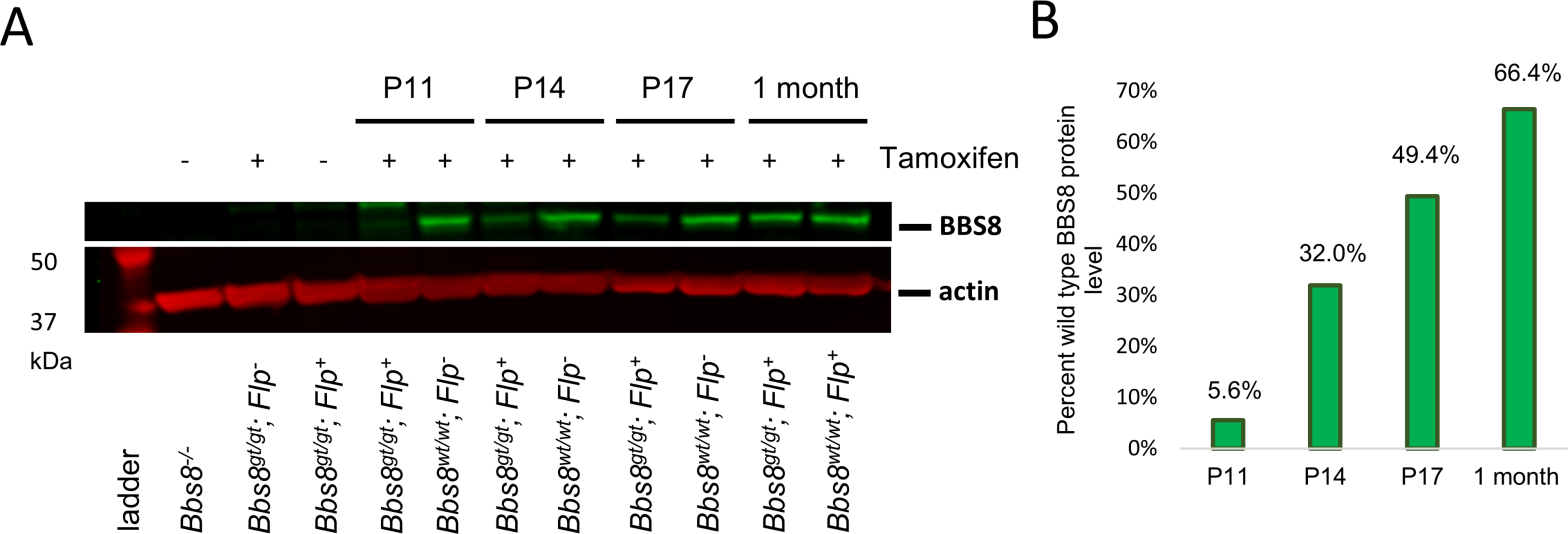
BBS8 protein levels in the eye during and after tamoxifen injections at P9, P12, and P15. (A) BBS8 protein levels in whole eye lysates of *Bbs8*^*gt/gt*^*; Flp*^*+*^ mice and *Bbs8*^*wt/wt*^*; Flp*^*+*^ or *Flp*^*-*^ mice. For the P11 time point, mice were injected with tamoxifen at P9 and sacrificed 2 days later. For the P14 time point, mice were injected at P9 and P12 and sacrificed 2 days later. For the P17 time point, mice were injected with tamoxifen at P9, 12, and 15 and sacrificed 2 days later. For the 1 month time point, mice were injected at P9, 12, and 15 and sacrificed 2 weeks later. (B) Percent BBS8 protein levels in *Bbs8*^*gt/gt*^*; Flp*^*+*^ mice compared to their respective wild type controls at those time points. BBS8 protein levels in the Western blot in panel (A) are quantified and normalized to actin. Normalized BBS8 protein levels of rescued mice are divided by normalized BBS8 protein levels of wild type mice to derive the ratio of BBS8 protein levels.

Because the timing of tamoxifen injections (P9, P12, and P15) is concurrent with the initial outer segment morphogenesis period (P9-P25), it was not clear whether the restoration of BBS8 proteins occurred in time to completely prevent outer segment malformation or whether the restoration of BBS8 protein occurred after the outer segment has already aberrantly formed. Therefore, we performed Western blot analysis to assess BBS8 protein levels in rescued mice at P11 (2 days after the 1^st^ tamoxifen injection), P14 (2 days after the 2^nd^ injection), and P17 (2 days after the 3^rd^ injection). We found that BBS8 protein levels in rescued mice are restored to 5.6%, 32.0%, and 49.4% of BBS8 protein levels in wild type littermates at P11, P14, and P17, respectively (Fig 12). Since BBS8 levels are lower than 50% of wild type levels prior to P17, it is unlikely that the restoration of BBS8 was in time to completely prevent outer segment malformation in rescued gene trapped mice.

**Fig 13:**
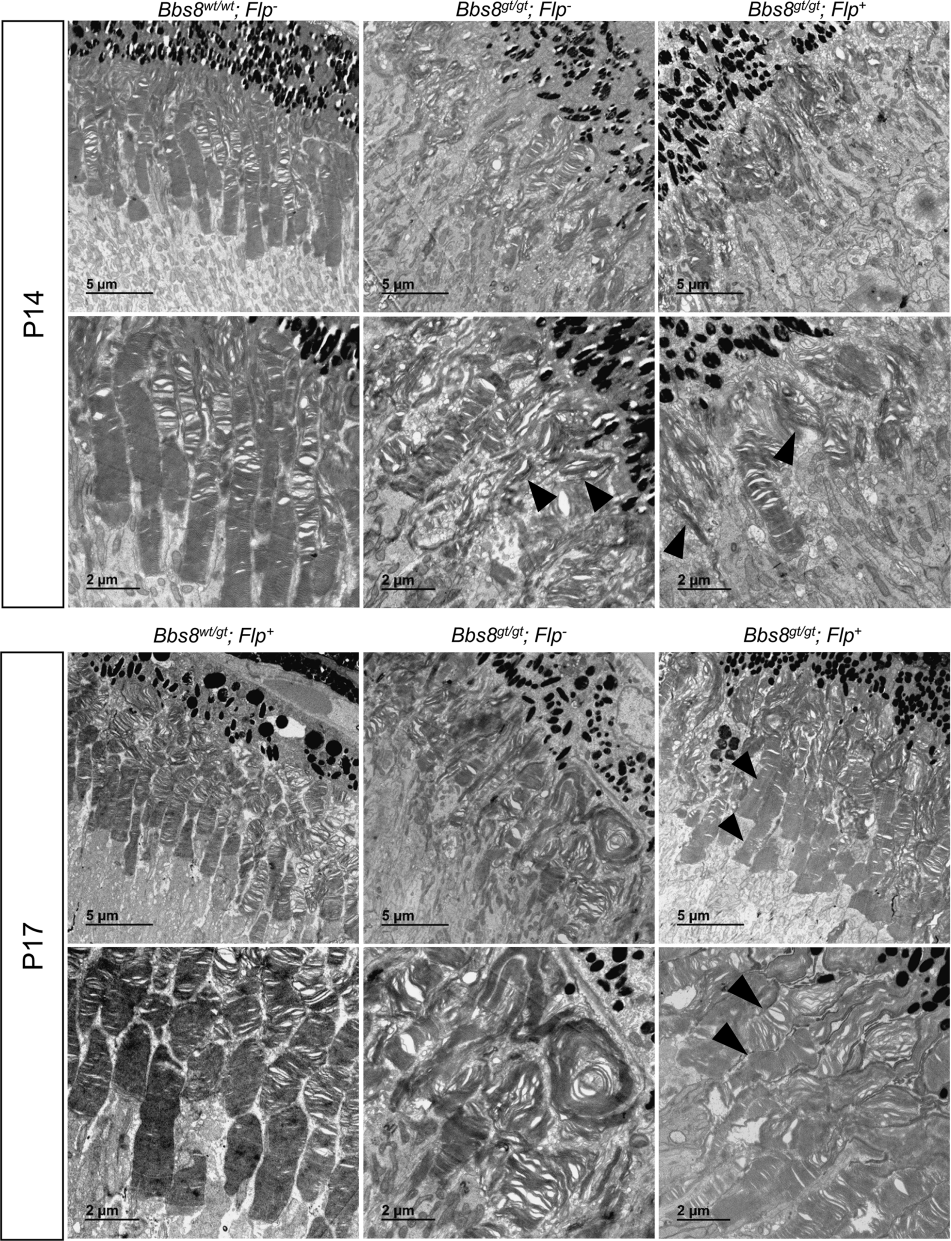
Outer segment morphology in rescued (*Bbs8*^*gt/gt*^*; Flp*^*+*^, tamoxifen treated) mice at P14 and P17. Outer segment morphology was examined by transmission electron microscopy at P14 (2 days after the 2^nd^ tamoxifen injection) and P17 (2 days after the 3rd tamoxifen injection). At P14, the majority of outer segments are still elongated and vertically oriented in rescued (*Bbs8*^*gt/gt*^*; Flp*^*+*^) mice similar to *Bbs8*^*gt/gt*^*; Flp*^*-*^ mice; arrowheads. At P17, numerous outer segments with disorganized and vertically oriented discs at the distal end but well-stacked discs at the proximal end can be observed in *Bbs8*^*gt/gt*^*; Flp*^*+*^ mice; arrowheads.

To determine how normal outer segment morphology was restored in rescued mice (Fig 11) if BBS8 levels at the onset of the outer segment morphogenesis period were insufficient, we performed TEM analysis at P14 and P17 to determine whether malformed outer segments were repaired, or whether they were displaced distally by normal outer segments as normal morphogenesis resumed. At P14 (2 days after the 2^nd^ tamoxifen injection), elongated and vertically oriented outer segments are observed in *Bbs8*^*gt/gt*^*; Flp*^*+*^ (rescued) mice, similar to those observed in *Bbs8*^*gt/gt*^*; Flp*^*-*^ mice (Fig 13), confirming that the restoration of BBS8 protein levels in rescued mice is too late to prevent the growth of malformed outer segments. However, at P17 (2 days after the last tamoxifen injection), we observed numerous outer segments with abnormal vertically oriented disc lamellae at their distal portion and normally oriented and tightly packed disc lamellae at their proximal portion (Fig 13, arrowheads, S14 Fig). These appear to represent outer segments that initially formed with misoriented discs that were displaced by properly formed discs after restoration of BBSome function. This phenomenon was not observed in treated *Bbs8*^*gt/gt*^*; Flp*^*-*^ mice at P17. In P17 control mice, the discs in the distal portion of the outer segments are more dilated compared to the proximal portion (Fig 13) as previously observed in P15 wild type mice (Fig 1B). Together, the Western blot and TEM data indicate that outer segments in rescued mice are initially formed abnormally and that restoration of BBSome function in immature retinas, even after initial outer segment malformation, enables the establishment of the normal outer segment morphogenesis process. For the rescue time point tested in this experiment, newly formed outer segments with normal morphology can gradually displace the malformed outer segments distally, resulting in the complete rescue of retinal morphology and function.

## Discussion

Measuring temporal protein expression provides insight into their biological role. By studying the expression of BBS proteins in wild type animals during eye development and maturation, we found that expression levels of both BBSome and non-BBSome components required for BBSome function (such as BBS3) are co-regulated. This regulation likely occurs at the mRNA level based on prior work using eQTL mapping to show highly correlated BBS gene mRNA levels [32]. The BBS protein expression profiles correspond to those of outer segment proteins ROM1 and PRPH2. All of these proteins have peak expression in the mouse eye at approximately P15, coinciding with the phase of outer segment development and rapid extension in mice. In mature eyes, at approximately 1 month of age, the levels of BBS proteins normalized to actin have dropped to about 50–75% of their respective peak levels. This suggests that the levels of BBS proteins needed to maintain mature eye function (i.e. regulating the compartmentalized protein distribution in the inner and outer segment and maintaining the structural integrity of the outer segment) are lower than the levels required during initial outer segment morphogenesis. In comparison to BBS proteins, IFT88 and IFT57, components of IFT complex B [30], reach their peak expression between P4 and P9, supporting the idea that IFT proteins and BBS proteins are required during distinct stages of photoreceptor development. IFT proteins are required for photoreceptor ciliogenesis and are localized to the mother centrioles, daughter centriole, and the ciliary vesicle during the ciliogenesis process [27]. The expression peak of IFT88 and IFT57 coincides with photoreceptor ciliogenesis in mice. For the proteins examined in this study, the expression profiles of proteins involved in outer segment formation versus those involved in ciliogenesis are distinct.

In cell culture and *in vivo*, the knockout of BBSome components does not abolish ciliogenesis [18, 33]. Even though ciliogenesis is not abolished in BBS mutant photoreceptors, their connecting cilia are elongated. We validated this observation with two different connecting cilia markers: anti-GT335 and anti-glycylated tubulin (TAP952). We provided further evidence for the expansion of the connecting cilia compartment by marking the upper and lower boundaries of the connecting cilia with RP1 and rootletin, respectively. It is worth noting that while this GT335 antibody recognizes glutamylated tubulin, it is also able to recognize glutamylated RPGR in the connecting cilia [34, 35], and that a significant portion of the GT335 signal in photoreceptor connecting cilia arises from glutamylated RPGR [35], admitting the possibility that the elongated glutamylation signal we observed could be due to glutamylated microtubules or RPGR. Abnormal ciliary length regulation such as elongation of photoreceptor cilia in male germ cell-associated kinase (*Mak*) mutant mice [36] is associated with outer segment malformation and retinal degeneration. Whether or not the elongation of connecting cilia in BBS partially underlies the pathogenic mechanism merits further investigation.

We observed outer segment morphogenesis defects in *Bbs8*^*-/-*^, *Bbs4*^*-/-*^, and *Bbs1*^*M390R/M390R*^ mice. We have previously demonstrated that there is no intact BBSome in the absence of BBS4 or BBS1 [9], and even though BBS1 and BBS4 are peripheral components of the BBSome [9], BBSome function is disrupted in the absence of these components [14, 19, 20]. Here, we confirm that there is no intact BBSome in the absence of BBS8 in tissues of *Bbs8*^*-/-*^ mice, and in sucrose gradient fractionation experiments, BBSome components are missing or shifted towards lower molecular weight fractions in the absence of BBS8. In eyes and testes of *Bbs8*^*-/-*^ mice, BBS2, BBS7, and BBS9 (considered the core of the BBSome) are found in fraction 9 to 11, whereas BBS1, BBS4, and BBS5 are missing or greatly reduced, suggesting that the core of the BBSome is still assembled in *Bbs8*^*-/-*^ mice. Immunoprecipitation of endogenous BBS2 shows preserved interaction with BBS7 and BBS9 in the absence of BBS8, which also supports the presence of the BBSome core consisting of BBS2, BBS7, and BBS9 in *Bbs8*^*-/-*^ mice. By comparison, in the absence of BBS1 or BBS4 the rest of the BBSome remain largely assembled [9], even though BBSome function is lost in the absence of these subunits [14, 19, 20]. We found that there is a lack of BBSome function in *Bbs8*^*-/-*^, *Bbs4*^*-/-*^, and *Bbs1*^*M390R/M390R*^ mice. Even though we cannot rule out possible differences in phenotypic severity between these mutants of BBSome components, all three mutant mouse models have outer segment morphogenesis defects, exhibit STX3 mislocalization to the outer segment, have low retinal function, and lose the majority of their photoreceptors by 6 months of age. We conclude that the absence of a functional BBSome causes these phenotypes.

The BBSome is required for protein trafficking in primary cilia. Outer segments are specialized structures for phototransduction, and the proper function of the outer segment requires the exclusion of non-outer segment proteins from this specialized compartment. Daily outer segment renewal calls for high directional flux of membrane materials from the inner to the outer segment, and the outer segment may act as a sink for membrane proteins [3]. The BBSome, either by acting as a gatekeeper or by retrieving proteins that aberrantly enters the outer segment, is needed to retain more than a hundred proteins in the inner segment, including STX3 [15]. We observed aberrant accumulation of STX3 in the outer segment in BBS mutant mice including *Bbs8*^*-/-*^, *Bbs4*^*-/-*^, and *Bbs1*^*M390R/M390R*^ during their initial outer segment morphogenesis (P15), confirming that the BBSome is required for establishing this compartmentalized protein distribution between the inner and outer segments. Additionally, when we deleted *Bbs8* after outer segments were normally formed, STX3 began to accumulate in the outer segment due to the loss of BBSome function. Together, these data illustrate that the BBSome prevents the accumulation of non-outer segment proteins in the outer segment during the initial formation of this structure, and continues to maintain this compartmentalized protein distribution throughout the life of the mature retina.

Despite differences in molecular mechanisms, BBS mutant mice share similar features with other mouse models with defective disc morphogenesis including the prominin-1 knockout [37], protocadherin-21 knockout [38], and peripherin-2 knockout (*Rds*^*-/-*^) mice [39–41]. Like BBS mutant mice, *Prom1*^*-/-*^ and *Pcdh21*^*-/-*^ mice are able to form connecting cilia, but fail to form or organize discs into mature outer segments. In *Rds*^*-/-*^ mice, outer segments do not form at all and disc membranes are instead released as ectosomes into the extracellular space [40]. Even though the molecular mechanisms differ, these models of defective disc morphogenesis share a similar rate of retinal degeneration. In these models, the progression of retinal degeneration leads to the loss of the majority of photoreceptor cells by 3–6 months of age, which resembles the rate of degeneration we observed in BBS mutant retinas. Mutations in genes that cause a defect in outer segment morphogenesis (without abolishing connecting cilia formation) may result in a similar rate of photoreceptor degeneration.

In contrast, the severity of retinal degeneration in BBS mutant mice differs significantly from that of IFT mutant mice. In cell culture, *Ift88* knockout cells have defective ciliogenesis [42]. Eye-specific knockout mouse models of *Ift88* or *Kif3a* using the *Six3-Cre* [26] have no observable connecting cilium formation, even though the site of supposed ciliogenesis (where the connecting cilium appears as a short stump) is correctly oriented in the apical domain. These eye-specific knockout mice have early onset retinal degeneration and lose the majority of their photoreceptors by P21, which is much faster than the rate of degeneration in BBS mutant mice.

The rate of degeneration in IFT mice is similar to other models with notable ciliogenesis defects. Recently, it has been reported that an eye-specific knockout model of *Macf1*, a protein that couples microtubules to the actin cytoskeleton, has both abnormal photoreceptor apico-basal polarization and ciliogenesis [25]. In this model, photoreceptors have defective orientation and formation of the connecting cilia, which are sometimes found within the outer nuclear layer as opposed to in the apical domain of photoreceptor cells. Not surprisingly, this model is characterized by disrupted lamination of the retina, and the majority of photoreceptors are lost by P21. Even though the structural integrity of both connecting cilia and outer segments are indispensable to photoreceptor health, it seems that mutations that abolish ciliogenesis lead to a significantly more severe retinal degeneration phenotype compared to mutations that cause only outer segment morphogenesis defects. As such, mutations in genes required for cilia formation cause a more severe phenotype (earlier onset and more rapid progression) compared to mutations in genes that are involved in later stages of retinal maturation, such as outer segment development and/or maintenance. Classification of retinal genes into these categories would facilitate the identification of potentially common rescue time windows for genes that act in the same pathway (i.e. outer segment formation), since identifying the rescue time window for every gene is impractical due to the extensive genetic heterogeneity of inherited eye diseases. Such classification would allow for knowledge gained derived from one gene to be applied to other genes acting in the same biological pathway, and would accelerate the design of intervention strategies for the genetically heterogeneous group of retinal disorders.

We restored BBSome function in postnatal *Bbs8*^*gt/gt*^*; Flp*^*+*^ mice by treating them with tamoxifen at P9, 12, and 15 to remove the gene trap inhibiting *Bbs8* expression. We observed morphologically and functionally normal retinas at 2 weeks and 4 months after injections. We initially thought that if BBS8 protein levels were restored rapidly after FLP-induced recombination, outer segment malformation may have been completely prevented in rescued mice. However, more detailed examination of the outer segment morphology at intermediate time points revealed that elongated and misoriented outer segments were already present in *Bbs8*^*gt/gt*^*; Flp*^*+*^ mice before BBSome function was restored. Upon the restoration of sufficient BBS8 levels, malformed outer segments were gradually displaced by normally formed outer segments. At P17 (two days after the last injection), we observed outer segments whose distal lamellae are abnormally elongated and vertically oriented but whose proximal lamellae have normal morphology characterized by horizontally stacked discs of normal diameter. These data suggest that correction of an outer segment morphogenesis defect remains possible even after outer segment malformation has already occurred if the correction takes place within a short window of photoreceptor plasticity. It is currently unknown how wide this window is. In the experiments described here, restoration of BBS8 protein levels was achieved prior to the full maturation of the retinas (which occurs around P25), when the outer segments were still undergoing active elongation. Whether the restoration of BBS8 in adult mice with malformed outer segments can completely rescue photoreceptor outer segment morphology is an open question that merits further investigation.

It is important to understand the developmental timeline in human eyes compared to mouse eyes in order to interpret any findings derived using the mouse as a model organism. In the parafovea of humans, short and immature inner and outer segments are present at birth, and these immature outer segments undergo rapid elongation during early postnatal months. Their full length is not reached until 2–5 years of age [23]. In the mid-peripheral region with high rod photoreceptor density, inner and outer segments are also short and exhibit immature morphology at birth, albeit slightly more developed than their counterparts in the parafovea, and continue to develop into early childhood [23].

The period of rapid outer segment growth in humans corresponds to approximately P9-P21 in mice when outer segments undergo rapid elongation before reaching their mature length and morphology by P21-P25 [1]. We show that restoration of BBSome function during the period of outer segment growth and elongation can fully rescue retinal phenotypes in mice. The P9, P12, and P15 time point described here approximately corresponds to a child less than two years of age. Due to the prevalence of polydactyly in BBS, the presence of this disorder can potentially be detected and molecularly diagnosed very early in children. This study suggests that administering treatment in the postnatal or early childhood period could have the potential of ameliorating retinal phenotypes.

Our current efforts are directed at investigating whether restoring BBSome function in remaining photoreceptor cells in older BBS mutant mice can arrest cell death and result in functional rescue in mid- to late- stages of retinal degeneration. This effort will elucidate whether there is a time window for the successful administration of gene replacement therapies, the degree of functional rescue attainable at each point of intervention, and suggest a clinically relevant transition from the administration of gene therapies to cell replacement therapies for the treatment of retinal degeneration. Together, these mechanistic insights lead to knowledge for the design of intervention strategies for the treatment of BBS-associated retinopathies as well as other retinopathies that primarily disrupt the morphogenesis of the photoreceptor outer segment.

## Materials and methods

### Ethics statement

This study was performed in strict accordance with the recommendations in the Guide for the Care and Use of Laboratory Animals of the National Institutes of Health. All of the animals were handled according to approved Institutional Animal Care and Use Committee (IACUC) protocols (#5061426 and #6081818) of the University of Iowa. Animals were housed according to IACUC recommendations. Methods of euthanasia used were carbon dioxide inhalation followed by cervical dislocation, or anesthesia induced by ketamine/xylazine followed by transcardial perfusion. Humane endpoints were strictly observed, and every effort was made to minimize suffering.

### Generation of the *Bbs8* congenital knockout line (*Bbs8*^*-/-*^)

All mouse genotypes listed in this manuscript are pre-tamoxifen genotypes. The *Bbs8* gene trap allele from the Knockout Mouse Project (KOMP) (*Ttc8*^*tm1a(KOMP)Wtsi*^, hereafter referred to as *Bbs8*^*gt*^) provides the ability to generate both floxed and deleted alleles from the original construct. We generated the *Bbs8* congenital knockout line (*Bbs8*^*-/-*^) by crossing mice with the *Bbs8*^*gt*^ allele to a ROSA26::FLPe knock in line (129S4/SvJaeSor-Gt(ROSA)26Sor^tm1(FLP1)Dym^/J, Jackson Lab #003946) to excise the gene trap. This generated a floxed allele, *Bbs8*^*flox*^. These mice were then bred to mice with an EIIa-Cre (B6.FVB-Tg(EIIa-cre)C5379Lmgd/J, Jackson Lab #003724) to induce a deletion of the floxed exons in germ line, resulting in the *Bbs8* congenital knockout allele (*Bbs8*^*-/-*^). In this manuscript, *Bbs8*^*-/-*^ refers to congenital knockout mice, *Bbs*^*gt/gt*^ refers to homozygous gene trap mice, and *Bbs8*^*flox/flox*^ refers to mice homozygous for the floxed *Bbs8* allele (S2 Fig).

### Immunoprecipitation

Wild type and mutant tissues were disrupted by TISSUEMISER (Fisher Scientific, Pittsburgh, PA) in lysis buffer (1X PBS, 0.8% Triton X-100 and protease inhibitor, Roche, Indianapolis, IN). The disrupted tissues were then sonicated three times, 15 s each, on ice. The lysates were then centrifuged at 20,000x*g* for 15 minutes and the concentrations of the supernatants were measured using the Bio-Rad Dc protein assay (Hercules, CA). 5 μg goat anti-BBS2 antibody (C-16, Santa Cruz Biotechnology, #sc-49381; Santa Cruz, CA) was added to 500 μL lysates which contained 1–1.5 mg total proteins and incubated overnight at 4°C, then 20 μL magnetic protein G beads were added to the lysates. The magnetic beads were washed four times with lysis buffer. Proteins were separated by electrophoresis using 4–12% NuPAGE Bis-Tris gels (Invitrogen, Carlsbad, CA) followed by transfer to nitrocellulose membranes and were detected by HyGLO chemiluminescent detection reagent (Denville Scientific, Holliston, MA).

### Sucrose gradient fractionation

Tissues were disrupted by TISSUEMISER in lysis buffer. The disrupted tissues were then sonicated three times, 15 s each, on ice. The lysates were centrifuged at 20,000x*g* for 20 minutes. The supernatants were loaded onto a 10–30% sucrose gradient prepared in lysis buffer. The gradient was centrifuged at 100,000x*g* for 16 hours using a TH-660 rotor (Thermo Scientific, Asheville, NC). Two hundred microliter fractions were taken from the top. Proteins were separated by electrophoresis using 4–12% NuPAGE Bis-Tris gels followed by transfer to nitrocellulose membranes and were detected by HyGLO chemiluminescent detection reagent. Anti-BBS1 and anti-BBS4 antibodies were generous gifts from Maxence Nachury at Standford University [8].

### Tamoxifen-inducible excision of *Bbs8*

*Bbs8*^*flox/flox*^ mice were crossed to *Bbs8*^*flox/-*^ mice to generate mice possessing one copy of the floxed *Bbs8* allele and one copy of the null allele for more efficient inactivation *Bbs8*. Offspring were generated with and without the tamoxifen-inducible CRE recombinase, B6.Cg-Tg(UBC-cre/ERT2)1Ejb/2J (Jackson Lab #008085). To activate the ERT2-Cre, adult *Bbs8*^*flox/-*^ mice were injected intraperitoneally with tamoxifen (Sigma Aldrich, St. Louis, MO) at a dose of 120 mg/kg (40 mg/mL in corn oil) beginning at two months of age for two sequential 5-day periods for a total of 10-day injections. Tamoxifen dose was adjusted daily by bodyweight. For the infantile cohort, both *Bbs8*^*flox/flox*^ and *Bbs8*^*flox/-*^ pups were injected subcutaneously with 40 μL (15 mg/mL in corn oil) of tamoxifen on three separate days (P9, P12, and P15). Efficient excision was achieved in both *Bbs8*^*flox/flox*^*; Cre*^*+*^ and *Bbs8*^*flox/-*^*; Cre*^*+*^ pups.

In order to assess excision efficiency, tail samples from tamoxifen-injected mice were obtained 1 week after the end of injections. After DNA extraction, PCR was performed using GoTaq (Promega, Madison, WI). PCR products were ran on 1.5% agarose gels along with a *Bbs8*^*flox/-*^*; Cre*^*-*^ control until the intensities of upper and lower bands from the *Bbs8*^*flox/-*^*; Cre*^*-*^ control were roughly equivalent (50 ± 5% of total intensity of upper and lower bands as measured by ImageJ). *Cre*^*+*^ animals determined to have less than 90% excision using this method were excluded from the study. No other outliers were excluded from the study except those excluded by the excision criteria described above.

### Genotyping

Mice were genotyped by PCR by using BioReady rTaq DNA polymerase (Bulldog Bio. Ins, Portsmouth, NH). Genomic DNA was extracted from tail snips. The 10 μL reaction includes 4 ng genomic DNA, 2 μM of each primer (listed in Table 1), and 0.2 mM dNTPs. All products were annealed at 55°C. Primers for genotyping FLP recombinase were designed by the Jackson Lab. The BBS8GT assay is used to detect the gene trap allele. The BBS8GN assay distinguishes the wild type allele from the *Bbs8* null allele. The BBS8MT assay distinguishes the unexcised floxed *Bbs8* allele from the null or excised *Bbs8* allele. The Cre assay tests for the presence or absence of *Cre*. The Flp assay tests for the presence or absence of *Flp*.

**Table 1:** List of genotyping primers.

PCR Assay	Primers	Composition	Sequences	Product Size
BBS8GT	F	33%	TGGCAAAGGTATGTGCTTCA	WT 257bp GT 168bp
	r1	33%	GTTGCGCCATCTCATAATAACTA	
	r2	33%	ACATACGCAGACAGTGTCAAGCT	
Flp	M	20%	TTATGTAACGCGGAACTCCA	WT 603bp FLP 309bp
	C	20%	AAAGTCGCTCTGAGTTGTTAT	
	W	60%	GGAGCGGGAGAAATGGATATG	
BBS8GN	f1	33%	TATCATCTTTGTAGGACGAAGCT	WT 193bp NULL 368bp
	f2	33%	AGAGTCATGTTAGAACTCCAAGT	
	R	33%	TGTCGGCTGCTGTCTGGTTGAGT	
BBS8MT	f1	33%	AGTTATTATGAGATGGCGCAACG	NULL/EXCISED 368bp FLOXED 250bp
	f2	33%	AGAGTCATGTTAGAACTCCAAGT	
	R	33%	TGTCGGCTGCTGTCTGGTTGAGT	
Cre	cre-f	10%	GGCCAGCTAAACATGCTTCATC	Ctrl 397bp Cre 250bp
	cre-r	10%	CCTGATCCTGGCAATTTCGGCT	
	ctrl-f	40%	CTGTACACCACGGTCTCCAAT	
	ctrl-r	40%	ATAATGCTGGGTGAAATGCAG	

The deletion efficiency of *Bbs8* exons by CRE recombinase and the excision efficiency of the *Bbs8*^*gt*^ allele by FLP recombinase were assessed by PCR using GoTaq (Promega) per the manufacturer’s recommendations. For assessing excision of the gene trap, products were amplified using a primer ratio of 2 Ttc8-FlpFrt2-Fr (5’ GAGTCATGTTAGAACTCCAAGT 3’): 1 Ttc8-FlpFrt2-Rv (5’ ACCACAAGGATAAGCCACACGT 3’): 1 Ttc8-UpFrt3-Rv (5’ TTGAAGGACTCCAATAGGGTAC 3’) and annealed at 55°C.

### Western blotting

Whole eyes were homogenized in lysis buffer (150 mM KCl, 2 mM MgCl_2_, 2 mM EGTA, 50 mM Hepes, 10% glycerol, 0.5 mM DTT) using a Dounce homogenizer. Homogenates were spun down at 10,000x*g* for 10 minutes, and the supernatant was aliquoted and stored at -80°C. Total protein concentration was determined by Bradford assay using DC Protein Assay (BioRad, Hercules, CA). Prior to loading, NuPage 10X reducing agent and NuPage 4X LDS Sample Buffer were added to the samples following the manufacturer’s instructions, and samples were heated for 5 minutes at 95°C. For Western blotting of BBS8, 100 μg of protein was loaded per lane, and lysate from a *Bbs8*^*-/-*^ animal was used as a negative control. Electrophoresis was performed using a 4–12% Bis-Tris denaturing gel and lysates were transferred onto nitrocellulose membranes overnight at 4°C. Membranes were incubated with blocking buffer (LI-COR Biosciences, Lincoln, NE) for an hour at room temperature and primary antibody incubation was performed overnight at 4°C in blocking buffer with 0.1% Tween20. Membranes were then washed three times with TBST containing 0.1% Tween20, followed by secondary antibody (LI-COR) incubation at room temperature for one hour. Following a second round of washes, imaging was performed using the LI-COR ODYSSEY CLx Imager and quantification of band intensities was performed using ImageJ (NIH, Bethesda, MD). The band intensities of target proteins were normalized to actin (loading control). For the time course Western blot of BBS8 protein levels at P11, P14, and P17, the BBS8 protein level in *Bbs8*^*gt/gt*^*; Flp*^*+*^ mice was divided by the BBS8 protein level in *Bbs8*^*wt/wt*^*; Flp*^*+*^ or *Flp*^*-*^ mice of the same age to derive the percentage of BBS8 protein restoration in rescued compared to wild type mice.

The antibodies used for Western blotting are as follows: BBS2 (Santa Cruz Biotechnology, #sc-49381, 1:500 dilution), BBS3 (Proteintech, #12676, 1:1000 dilution), BBS5 (SantaCruz Biotechnology, #515331, 1:500 dilution), BBS7 (Proteintech, #18961, 1:500 dilution), BBS8 (Sigma-Aldrich #HPA003310, 1:500 dilution), prominin-1 (Thermo Fisher, #PA5-38014, 1:750 dilution), IFT88 (Proteintech, #13967, 1:1000 dilution), IFT57 (Proteintech, #11083, 1:1000 dilution), MACF1 (Proteintech, #13058, 1:1000 dilution), actin (rabbit, Sigma-Aldrich, #A5060, 1:2000 dilution; mouse, Abcam, #6276, 1:3000 dilution). IFT57 over expression lysate used as a positive control was purchased from Origene, Rockville, MD (#LC402637).

### Electroretinography (ERG)

Mice were dark adapted overnight. Under red light, mice were anesthetized by intraperitoneal injection with Ketamine (17.5 mg/cc)/Xylazine (2.5 mg/cc) (100 μL/20 g body weight). After anesthesia, the eye was dilated for 5 minutes through application of Phenylephrine HCl (Paragon, Portland, OR). Recordings (10 sweeps per intensity, 1 ms per sweep, green light) were made at 0.1564, 1.252, and 40 cd·sec/m^2^ using the ERG system (Phoenix Research Labs, Pleasanton, CA) and LabScribe2 ERG software. The a- and b-wave amplitudes reported as bar graphs in this study are combined response scotopic ERG using light intensity of 1.252 cd·sec/m^2^. After recordings, animals were allowed to recover on a heat pad per IACUC recommendations.

### Histology and immunohistochemistry

Mice were anesthesized by intraperitoneal ketamine and xylazine mixture as previously described and transcardial perfusion was performed using 10% paraformaldehyde at 2.5 ml/min for a total volume of 1.25 mL/g body weight. Eyes were enucleated, and a small puncture was created through the lens using a 26-G syringe. Eyes were then embedded in Tissue-Tek O.C.T. compound (VWR, Batavia, IL) and frozen in 2-methybutane chilled with liquid nitrogen. Eyes were sectioned using a Cryostat microtome at thickness of 10 microns followed by hematoxylin and eosin staining. The quantification of retinal thickness was performed by an experimenter masked to genotypes.

For syntaxin-3 immunohistochemistry, sections were permeabilized with 0.3% Triton X-100 in PBS, blocked in a blocking buffer containing 5% BSA, 5% Normal Goat Serum, and 0.05% Triton X-100 in PBS, and incubated with primary antibodies at 4°C overnight. The next day, slides were washed three times followed by secondary antibody incubation at room temperature for 1 hour. After washing slides were mounted with VectaShield anti-fade mounting medium with DAPI (Vector Laboratories, Burlingame, CA). Images were taken using a fluorescence microscope.

For immunohistochemistry of rootletin, GT335, glycylated tubulin, RP1, PALS1, and β-catenin, animals were euthanized with CO_2_ followed by cervical dislocation without transcardial perfusion. Eyes were enucleated, a small puncture was created through the lens with a 26-G syringe, and embedded in Tissue-Tek O.C.T. medium without fixation. These blocks were frozen in a 2-methylbutane bath chilled with liquid nitrogen. Sections were prepared at 10 micron thickness, and slides were fixed in 4% paraformaldehyde in PBS for 10 minutes at 4°C prior to permeabilization and primary antibody incubation. For deriving the ciliogenesis frequency, the number of connecting cilia per a defined length of the retina were quantified by an experimenter masked to genotypes. At least three images were analyzed per animal. The quantification of the length of GT335 and glycylated tubulin (TAP952) signal in the connecting cilium was performed in ImageJ by an experimenter masked to genotypes. The analysis of connecting cilia length is presented in two ways. In scatter plots, each data point represents one connecting cilium, and the plot shows 30 connecting cilia from one knockout mouse with its control littermate. The bar graphs represent averaged values from three control and three knockout mice, where 30 connecting cilia from each animal were averaged and considered as a single datapoint representing connecting cilia length of that animal. Both types of graphs are shown to demonstrate both length distribution of connecting cilia in an animal and reproducibility of the results in different animals.

The antibodies used for immunohistochemistry are as follows: MPP5/PALS1 (Proteintech, Rosemont, IL, #17710, 1:1000 dilution), β-catenin (Transduction Laboratories/BD Biosciences, San Jose, CA, #610154, 1:1000 dilution), GT335 (Adipogen, San Diego, CA, #AG-20B-0020-C100, 1:1000 dilution), rootletin (Millipore, Billerica, MA, #ABN1686, 1:400 dilution), TAP952 (Millipore, #MABS277, 1:200), syntaxin-3 (Proteintech, #155556, 1:1000 dilution), RP1 (Bethyl Laboratories, Montgomery, TX, #A302-713A, 1:100 dilution), rhodopsin (Santa Cruz Biotechnology, Dallas, TX, #sc-57432, 1:1000 dilution).

### Transmission electron microscopy (TEM)

Transmission electron microscopy was performed as described elsewhere with modifications [43]. Animals were anesthetized with the ketamine/xylazine mixture as previously described and perfused transcardially with fixative containing 2% paraformaldehyde, 2% glutaraldehyde, and 0.05% CaCl_2_ in 50 mM MOPS buffer, pH 7.4 [44]. Eyes were immediately enucleated and eye-cups were harvested by dissecting out the anterior segment and the lens. Eye-cups were further incubated in this fixative for one hour at 4°C. Eye-cups were then transferred to a new fixative solution containing 2% paraformaldehyde and 2% glutaraldehyde in 0.15 M sodium cacodylate buffer and were incubated overnight at 4°C. The fixed eyecups were stained with 1% OsO_4_ containing 1.5% potassium ferrocyanide and further contrasted with 2.5% uranyl acetate. Samples were dehydrated using increasing concentrations of ethanol and propylene oxide (Sigma-Aldrich), infiltrated, embedded with Eponate 12 resin (Ted Pella, Redding, CA), and polymerized in a 70°C oven. Ultrathin sections (70–90 nm) were cut with a Leica UC6 ultramicrotome (Leica Microsystems). Sections were collected on copper grids coated with the Formvar (Ted Pella) and sequentially stained with 5% uranyl acetate and Reynold’s lead citrate. The retinas were viewed using a Jeol 1230 transmission electron microscope (Jeol USA, Peabody, MA) equipped with a Gatan Ultrascan 2k × 2k CCD camera. All used reagents were electron microscopy grade (Electron Microscopy Sciences, Hatfield, PA).

### Power analysis, sample size, and statistical analysis

We performed a longitudinal ERG study (S7 Fig) at different ages in *Bbs8*^*-/-*^ mice to establish typical ERG responses in mutant mice. We then performed power analysis using the Z-test method to estimate the sample sizes required for detecting changes in ERG responses after CRE-mediated deletion of *Bbs8* in infantile mice, with the assumption that the effect size would be smaller and variability greater in tamoxifen-inducible mice compared to congenital knockout mice. For example, based on the averages of ERG a-wave amplitudes at one month of age (control: -100.8, knockout: -27.6 μV) and their standard deviations (control: 22.2, knockout: 11.0 μV), we estimated that the ERG responses in 1 month old tamoxifen-treated mice in the infantile cohort would be approximately -100 and -70 μV for *Cre*^*-*^ controls and *Cre*^*+*^ mice, respectively, and the standard deviation would be approximately 25 μV because of the expected variability in CRE-mediated excision efficiency compared to a congenital knockout mouse model. Using these assumptions, the estimated sample size for each group was 13 for a power of 0.95 and a 99% confidence in the observation. Sample sizes for the adult cohort were calculated based on experimental values derived from the infantile cohorts, and were smaller due to the existence of this knowledge. The sample sizes for other experiments were calculated using similar methods. In this study, each animal represents a biological replicate and is considered a single data point.

Error bars in figures represent standard error of the mean (SEM). Welsh’s test, a modification of the Student *t*-test with assumption of unequal sample variances, was performed for single comparisons. Two-tail p values are reported. For multiple comparisons, one way ANOVA was performed followed by post-hoc Tukey’s test. Analyses were performed using GraphPad PRISM software.

## Supporting information

S1 Fig Temporal expression profiles of BBS and IFT proteins in postnatal eye. Each lane contains whole eye lysates from two wild type mice. Eye lysates from knockout mice of indicated proteins or overexpression cell lysates were used as negative and positive controls. The *Bbs7*^*-/-*^*; Bbs8*^*-/-*^ double knockout eye lysate was derived from a one year old mouse with few or no photoreceptors remaining, and this lysate served as a negative control for PRPH2 and MACF1.
(TIF)

S2 Fig The *Bbs8* gene trap allele for the generation of *Bbs8*^*-/-*^, *Bbs8*^*flox/flox*^, and *Bbs8*^*gt/gt*^ mouse models. (A) The original KOMP allele (*Ttc8*^*tm1a(KOMP)Wtsi*^, herein referred to as *Bbs8*^*gt*^). (B) The *Bbs8*^*flox*^ allele. (C) The *Bbs8* null allele.
(TIF)

S3 Fig Disassembly of the BBSome in eyes and testes of *Bbs8*^*-/-*^ mice. (A) Immunoprecipitation of BBSome components by endogenous BBS2 in eyes. (B) Sucrose gradient fractionation of eye lysates of wild type and *Bbs8*^*-/-*^ mice. (C) Immunoprecipitation of BBSome components by endogenous BBS2 in testes. (D) Sucrose gradient fractionation of testes lysates of wild type and *Bbs8*^*-/-*^ mice. In sucrose gradient figures, the fraction where the BBSome components are observed is marked with an arrow, whereas the protein signal is marked with an arrowhead.
(TIF)

S4 Fig Quantification of the thicknesses of retinal layers in P15 *Bbs8*^*-/-*^ mice and in *Bbs1*^*M390R/M390R*^ mice. Thicknesses of retinal layers in H and E histological sections were quantified with ImageJ by an experimenter masked to genotypes. At this age, both mutants have shortened inner and outer segments compared to their control littermates, and a mild but statistically significant reduction in the thickness of the outer nuclear layer.
(TIF)

S5 Fig Localization of PALS1, β-catenin, and GT335 in P15 *Bbs8*^*-/-*^ mice. (Top) PALS1 localizes to the apical domain of photoreceptor cells in both control and *Bbs8*^*-/-*^ mice. PALS1 was co-stained with β-catenin. (Bottom) Enlarged images of PALS1 localization and connecting cilia marked by GT335 in control and *Bbs8*^*-/-*^ photoreceptors.
(TIF)

S6 Fig Ciliogenesis frequency in P15 control and *Bbs8*^*-/-*^ retinas. The number of connecting cilia as labeled by anti-GT335 antibodies per 10 μm length of retina was quantified by an experimenter masked to genotypes. P value is shown for two-tailed *t*-test.
(TIF)

S7 Fig Longitudinal study of ERG responses in *Bbs8*^*-/-*^ mice. P values from two-tailed *t-*test are shown for each age, where * denotes p < 0.05, ** denotes p < 0.01, *** denotes p < 0.005, and **** denotes p < 0.001.
(TIF)

S8 Fig Comparison of retinal function in *Bbs8*^*-/-*^, *Bbs4*^*-/-*^, and *Bbs1*^*M390R/M390R*^ mice at 6–7 months of age. At this age, ERG responses of *Bbs8*^*-/-*^ and *Bbs4*^*-/-*^ were non-recordable, whereas the ERG responses of *Bbs1*^*M390R/M390R*^ were extremely low containing a residual waveform. However, there was no statistically significant difference between the a- or b-wave amplitudes of *Bbs8*^*-/-*^, *Bbs4*^*-/-*^, and *Bbs1*^*M390R/M390R*^ mice. P values from one-way ANOVA followed by post-hoc Tukey’s test are shown.
(TIF)

S9 Fig Comparison of retinal degeneration in *Bbs8*^*-/-*^, *Bbs4*^*-/-*^, and *Bbs1*^*M390R/M390R*^ mice at 6–7 months of age. H and E histological sections are shown for *Bbs8*^*-/-*^, *Bbs4*^*-/-*^, and *Bbs1*^*M390R/M390R*^ mice at 6–7 months of age. Thicknesses of retinal layers in 6–7 months old *Bbs8*^*-/-*^ mice were quantified in ImageJ by an experimenter masked to genotypes. P values from two-tailed *t-*tests are shown.
(TIF)

S10 Fig Reduction in BBS8 protein levels only occurs after tamoxifen (TMX) injections. BBS8 levels were normalized to actin as a loading control. There is no reduction in BBS8 protein in *Cre*^*+*^, uninjected mice. After tamoxifen injections, BBS8 protein levels were reduced by approximately 80% compared to their controls. Of note, the BBS8 protein level in control *Bbs8*^*flox/-*^*; Cre*^*-*^ mice, where there is only one copy of *Bbs8* allele, is approximately 90% of that in *Bbs8*^*flox/flox*^*; Cre*^*-*^ control mice with two *Bbs8* alleles, suggesting that there may be BBS8 protein level compensation in *Bbs8* heterozygotes.
(TIF)

S11 Fig CRE induction by tamoxifen in the presence of the *Bbs8* wild type allele does not cause retinal degeneration. *Bbs8*^*wt/flox*^ *or Bbs8*^*wt/wt*^*; Cre*^*+*^ mice were treated with tamoxifen (TMX) between P9-15 along with *Bbs8*^*flox/flox*^ or *Bbs8*^*flox/-*^*; Cre*^*-*^ mice and *Bbs8*^*flox/flox*^ *or Bbs8*^*flox/-*^*; Cre*^*+*^ mice. Histology and ERG were performed 6–7 months after the last tamoxifen injection. Thicknesses of retinal layers were quantified in ImageJ by an experimenter masked to genotypes. No adverse effect of CRE activation was observed in *Bbs8*^*wt/flox*^ *or Bbs8*^*wt/wt*^*; Cre*^*+*^ mice. Retinal degeneration was only observed in *Bbs8*^*flox/flox*^ *or Bbs8*^*flox/-*^*; Cre*^*+*^ mice. P values reported are derived from one-way ANOVA followed by post-hoc Tukey’s test for multiple comparisons.
(TIF)

S12 Fig Retinal degeneration in adult (top) and infantile (bottom) cohorts 4 months after the last tamoxifen injection. *Bbs8*^*flox/flox*^ *or Bbs8*^*flox/-*^*; Cre*^*-*^ or *Cre*^*+*^ mice were treated with tamoxifen (TMX) and retinal histology was performed 4 months after the last TMX injection. Thicknesses of retinal layers were quantified in ImageJ by an experimenter masked to genotypes. P values reported are derived from two-tailed *t-*test.
(TIF)

S13 Fig Retinal degeneration in adult (top) and infantile (bottom) cohorts 7 months after the last tamoxifen injection. *Bbs8*^*flox/flox*^ *or Bbs8*^*flox/-*^*; Cre*^*-*^ or *Cre*^*+*^ mice were treated with tamoxifen (TMX) and retinal histology was performed 7 months after the last injection. Thicknesses of retinal layers were quantified in ImageJ by an experimenter masked to genotypes. P values reported are derived from two-tailed *t-*test.
(TIF)

S14 Fig Outer segment ultrastructure in *Bbs8*^*gt/gt*^*; Flp*^*+*^, tamoxifen (TMX)-treated mice at P17. Two days after the 3^rd^ tamoxifen injection (P9, P12, P15), outer segment ultrastructure was examined by transmission electron microscopy. Outer segments with distally disorganized discs that are vertically oriented, but proximally organized discs that are horizontally stacked are observed in these rescued mice.
(TIF)
