# Supplementary figures and images for: BBSome function is required for both the morphogenesis and maintenance of the photoreceptor outer segment
Source: PLoS Genet. 2017 Oct 19;13(10):e1007057. doi: 10.1371/journal.pgen.1007057 (PMC5663628; doi:10.1371/journal.pgen.1007057)

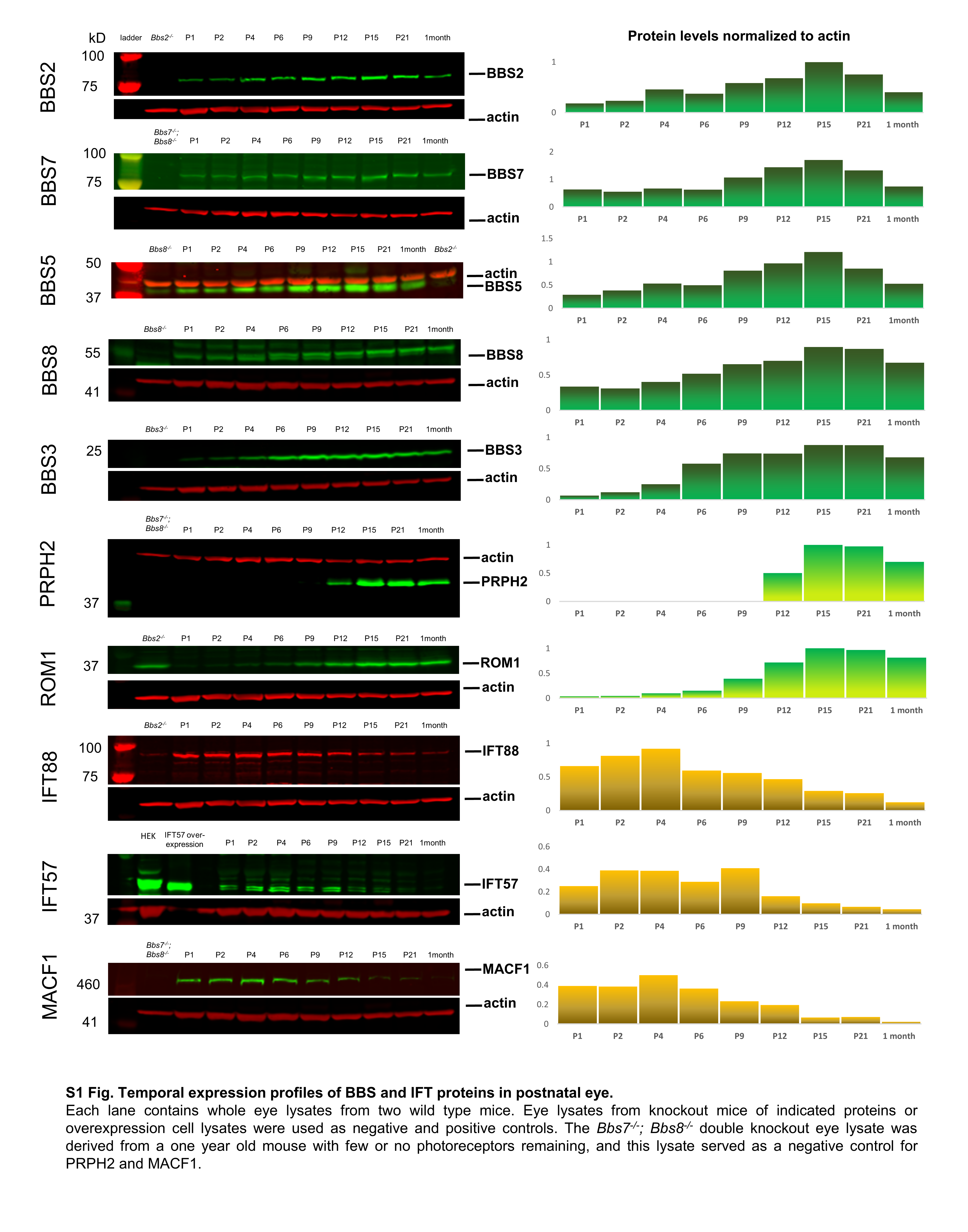

Supplement: S1 Fig — Each lane contains whole eye lysates from two wild type mice. Eye lysates from knockout mice of indicated proteins or overexpression cell lysates were used as negative and positive controls. The Bbs7-/-; Bbs8-/- double knockout eye lysate was derived from a one year old mouse with few or no photoreceptors remaining, and this lysate served as a negative control for PRPH2 and MACF1. (TIF) [file pgen.1007057.s001.TIF]

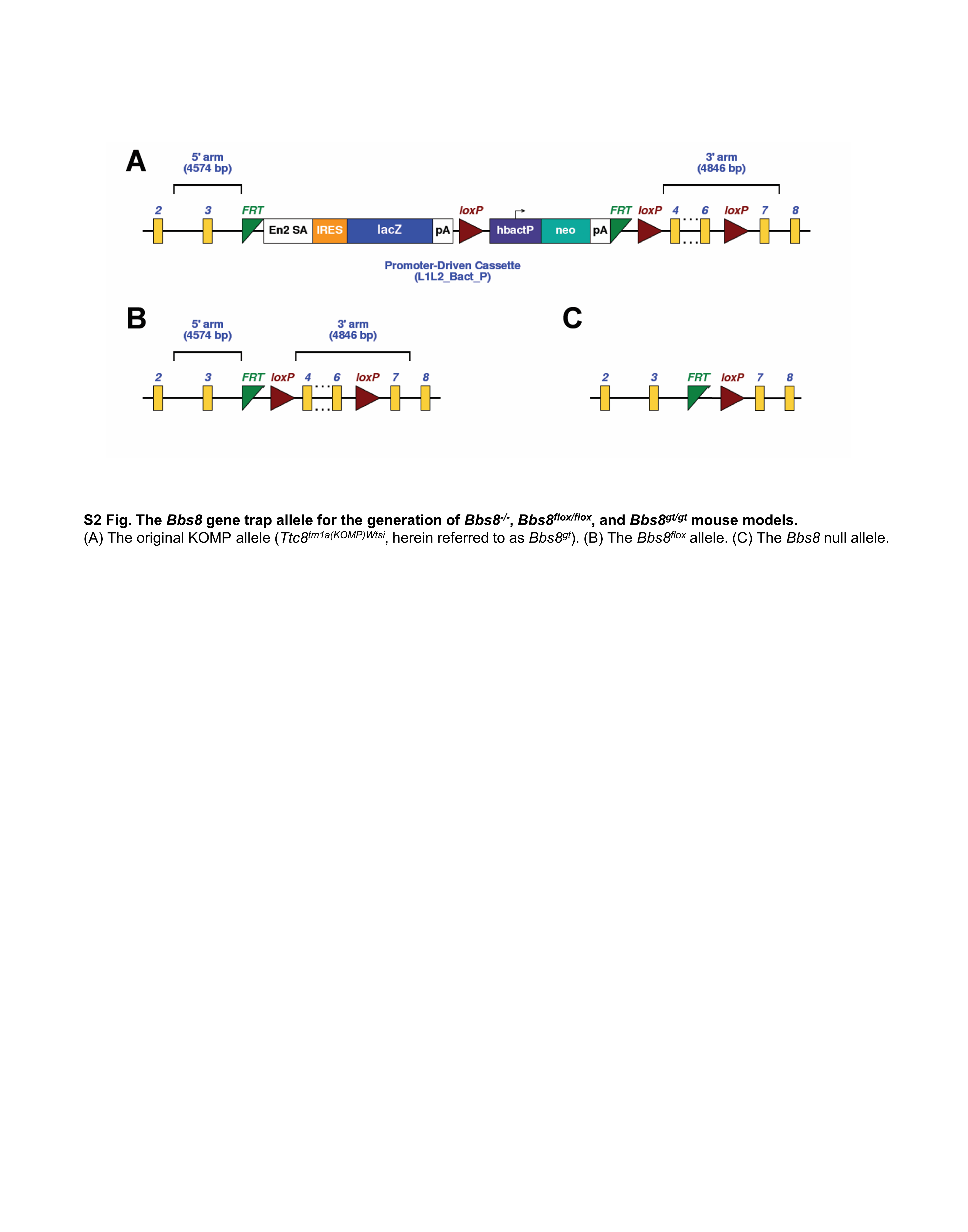

Supplement: S2 Fig — (A) The original KOMP allele (Ttc8tm1a(KOMP)Wtsi, herein referred to as Bbs8gt). (B) The Bbs8flox allele. (C) The Bbs8 null allele. (TIF) [file pgen.1007057.s002.TIF]

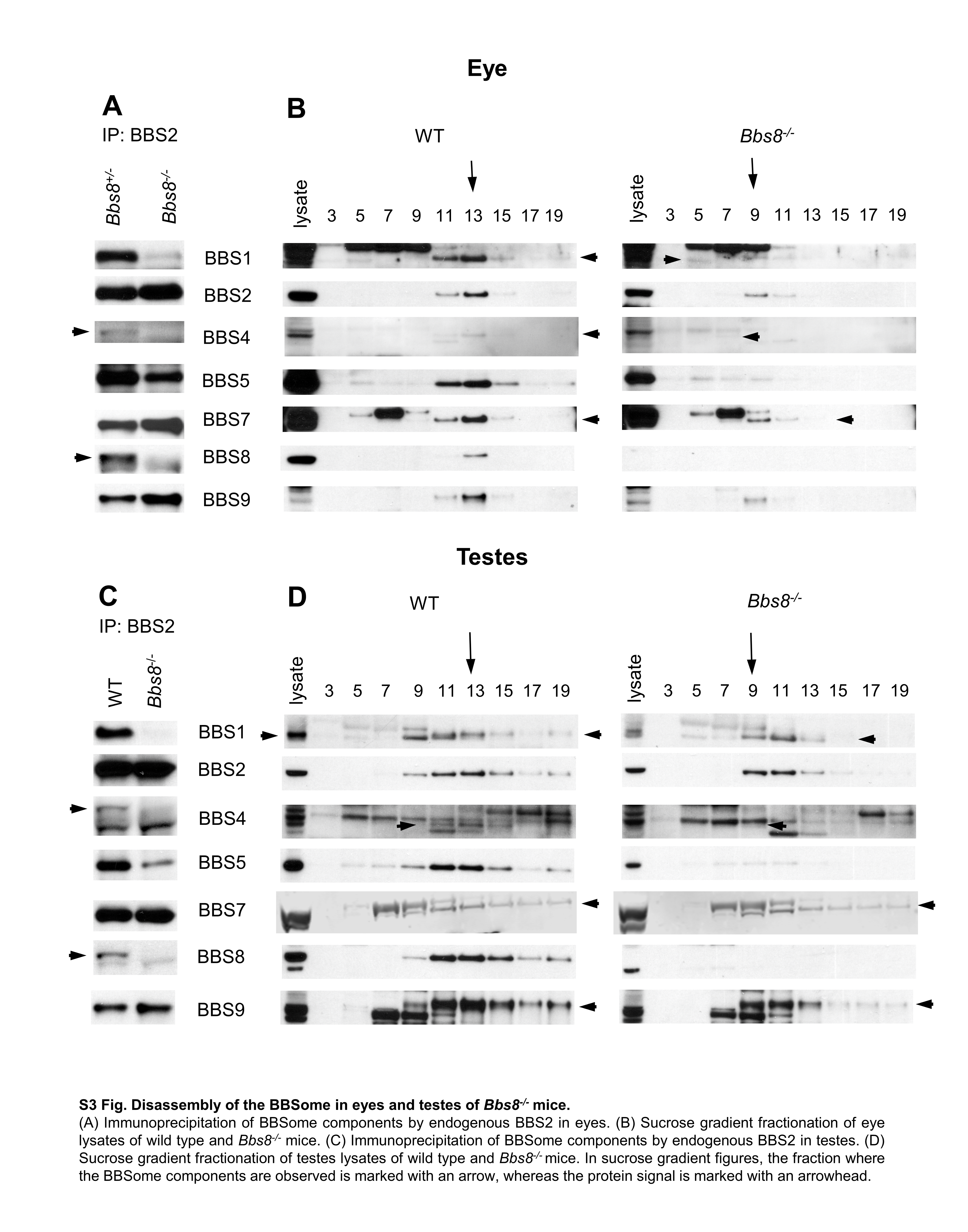

Supplement: S3 Fig — (A) Immunoprecipitation of BBSome components by endogenous BBS2 in eyes. (B) Sucrose gradient fractionation of eye lysates of wild type and Bbs8-/- mice. (C) Immunoprecipitation of BBSome components by endogenous BBS2 in testes. (D) Sucrose gradient fractionation of testes lysates of wild type and Bbs8-/- mice. In sucrose gradient figures, the fraction where the BBSome components are observed is marked with an arrow, whereas the protein signal is marked with an arrowhead. (TIF) [file pgen.1007057.s003.TIF]

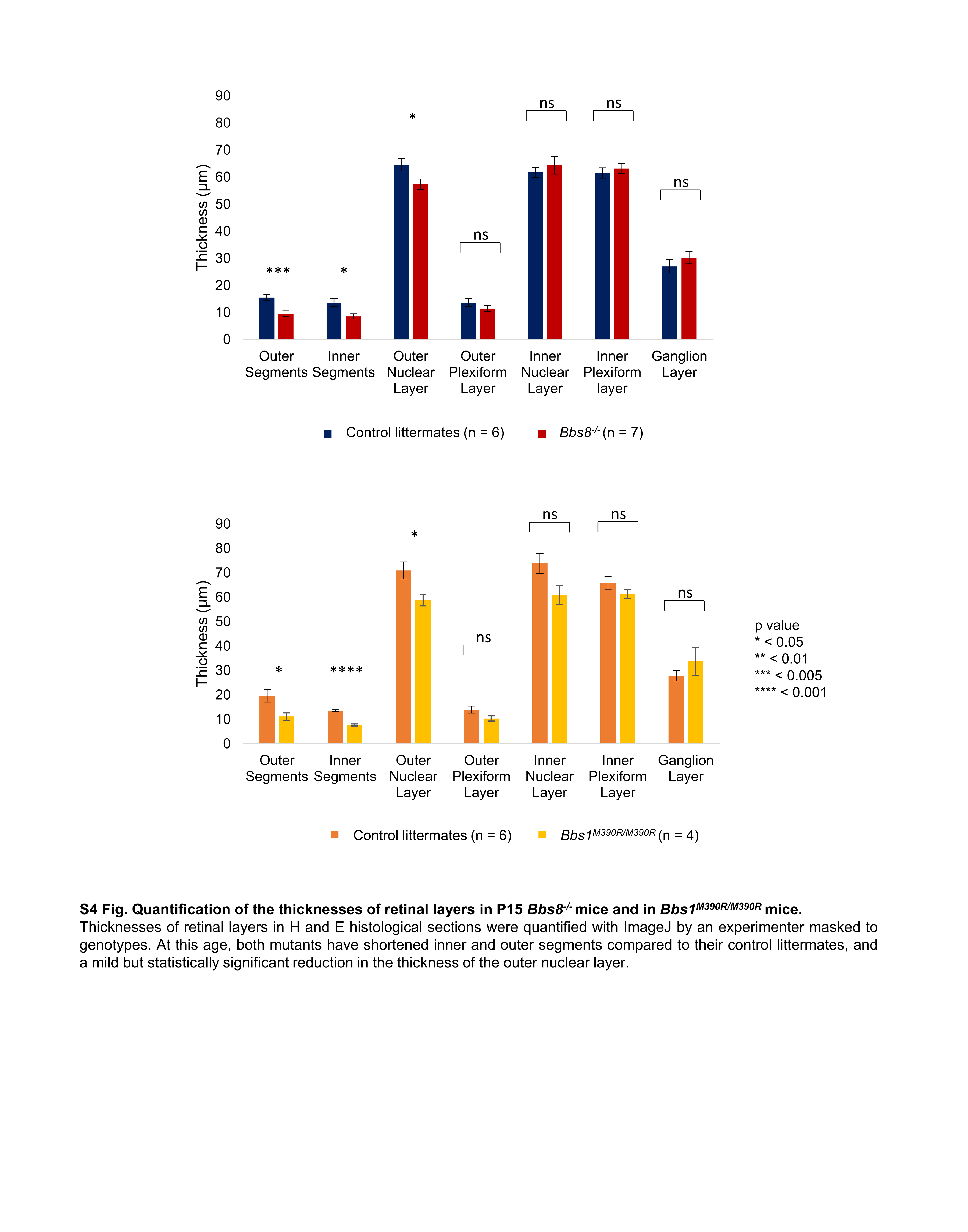

Supplement: S4 Fig — Thicknesses of retinal layers in H and E histological sections were quantified with ImageJ by an experimenter masked to genotypes. At this age, both mutants have shortened inner and outer segments compared to their control littermates, and a mild but statistically significant reduction in the thickness of the outer nuclear layer. (TIF) [file pgen.1007057.s004.TIF]

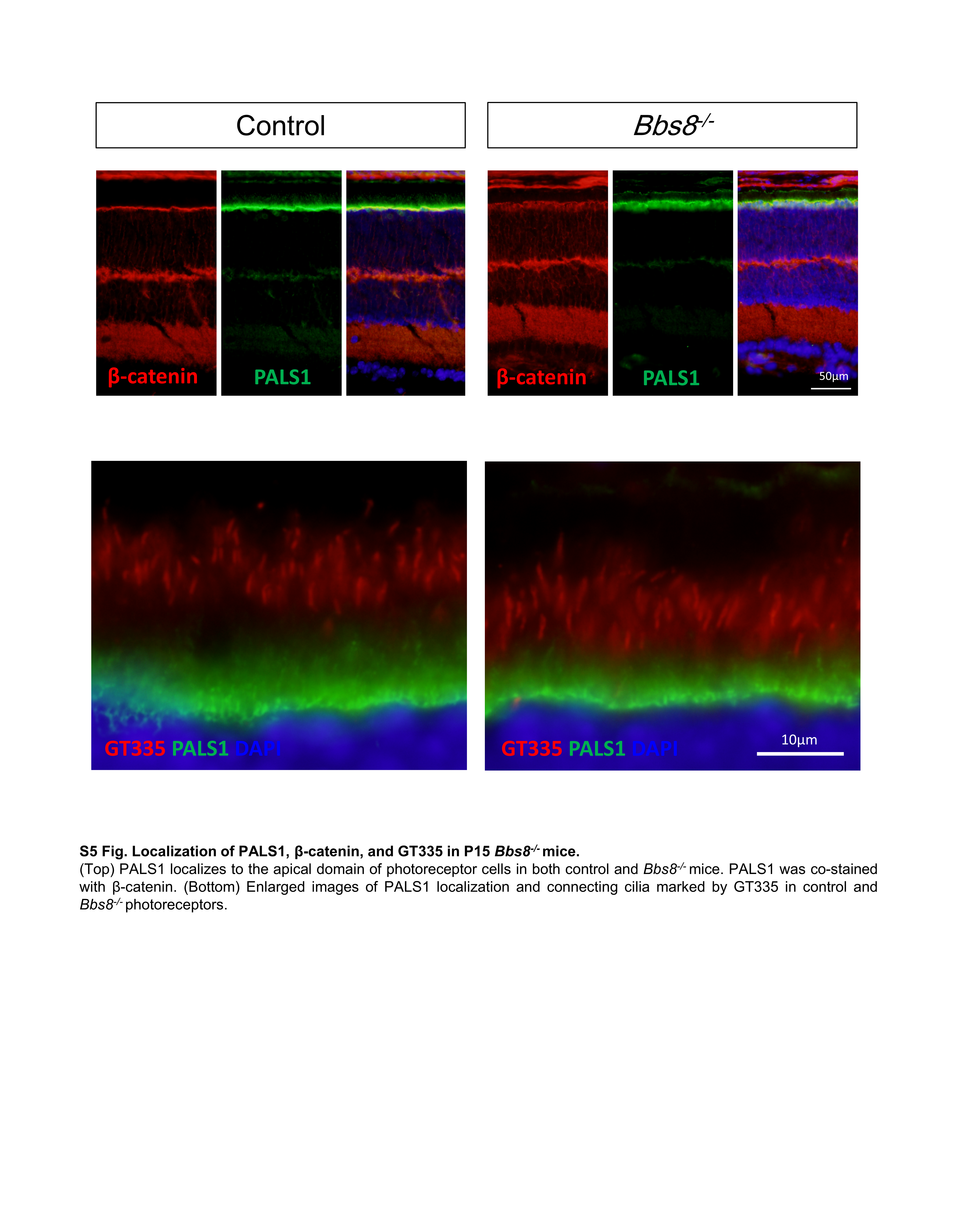

Supplement: S5 Fig — (Top) PALS1 localizes to the apical domain of photoreceptor cells in both control and Bbs8-/- mice. PALS1 was co-stained with β-catenin. (Bottom) Enlarged images of PALS1 localization and connecting cilia marked by GT335 in control and Bbs8-/- photoreceptors. (TIF) [file pgen.1007057.s005.TIF]

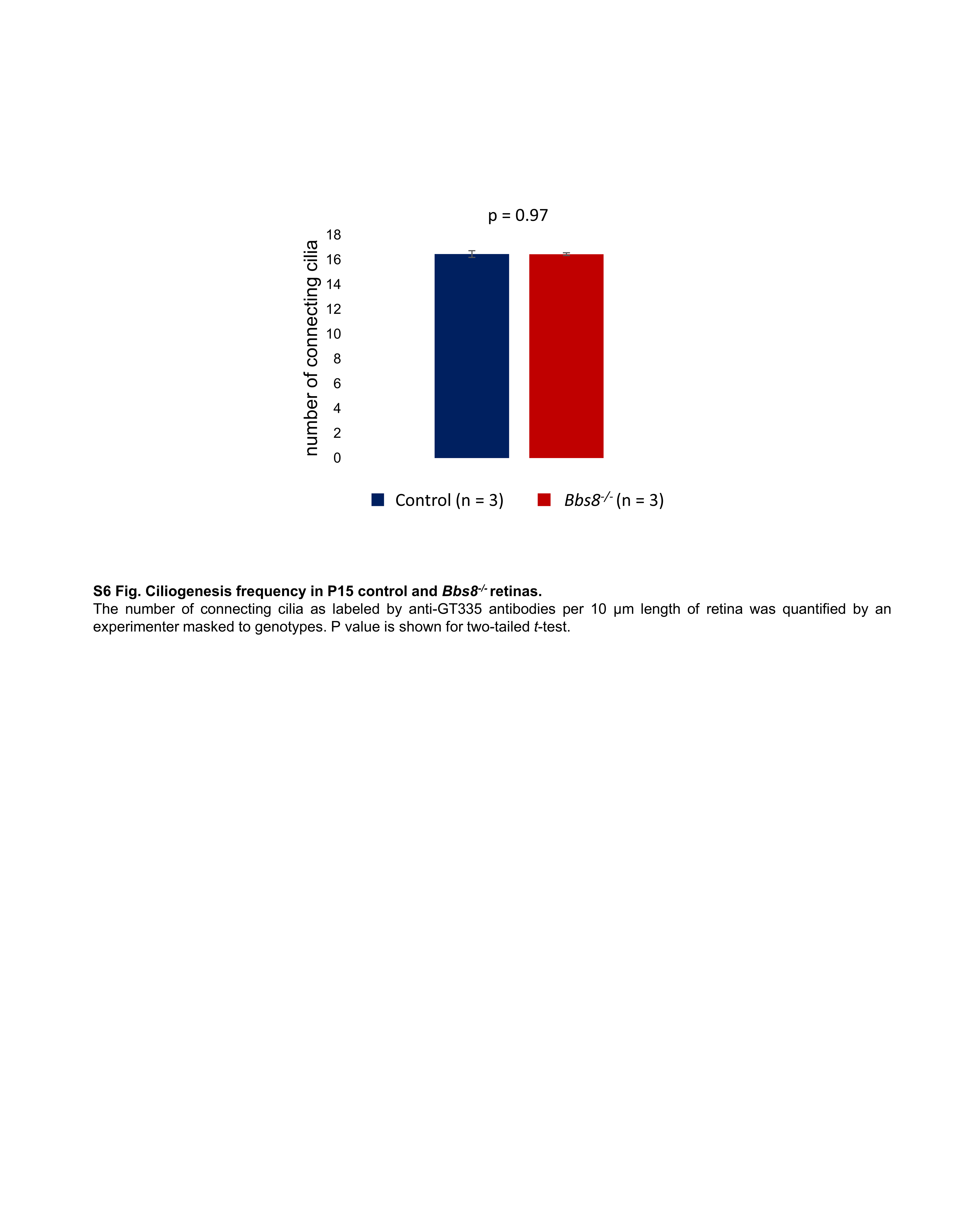

Supplement: S6 Fig — The number of connecting cilia as labeled by anti-GT335 antibodies per 10 μm length of retina was quantified by an experimenter masked to genotypes. P value is shown for two-tailed t-test. (TIF) [file pgen.1007057.s006.TIF]

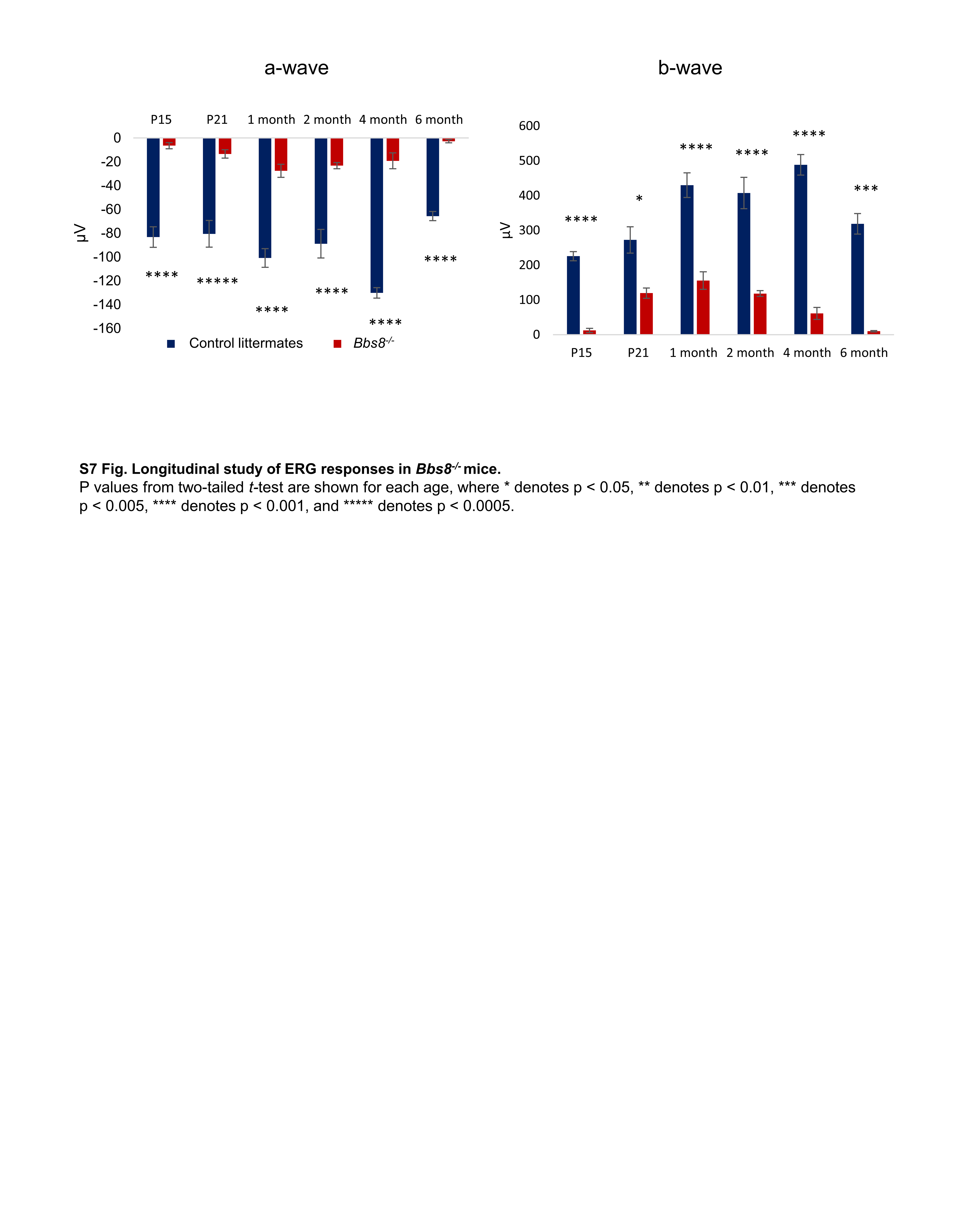

Supplement: S7 Fig — P values from two-tailed t-test are shown for each age, where * denotes p < 0.05, ** denotes p < 0.01, *** denotes p < 0.005, and **** denotes p < 0.001. (TIF) [file pgen.1007057.s007.tif]

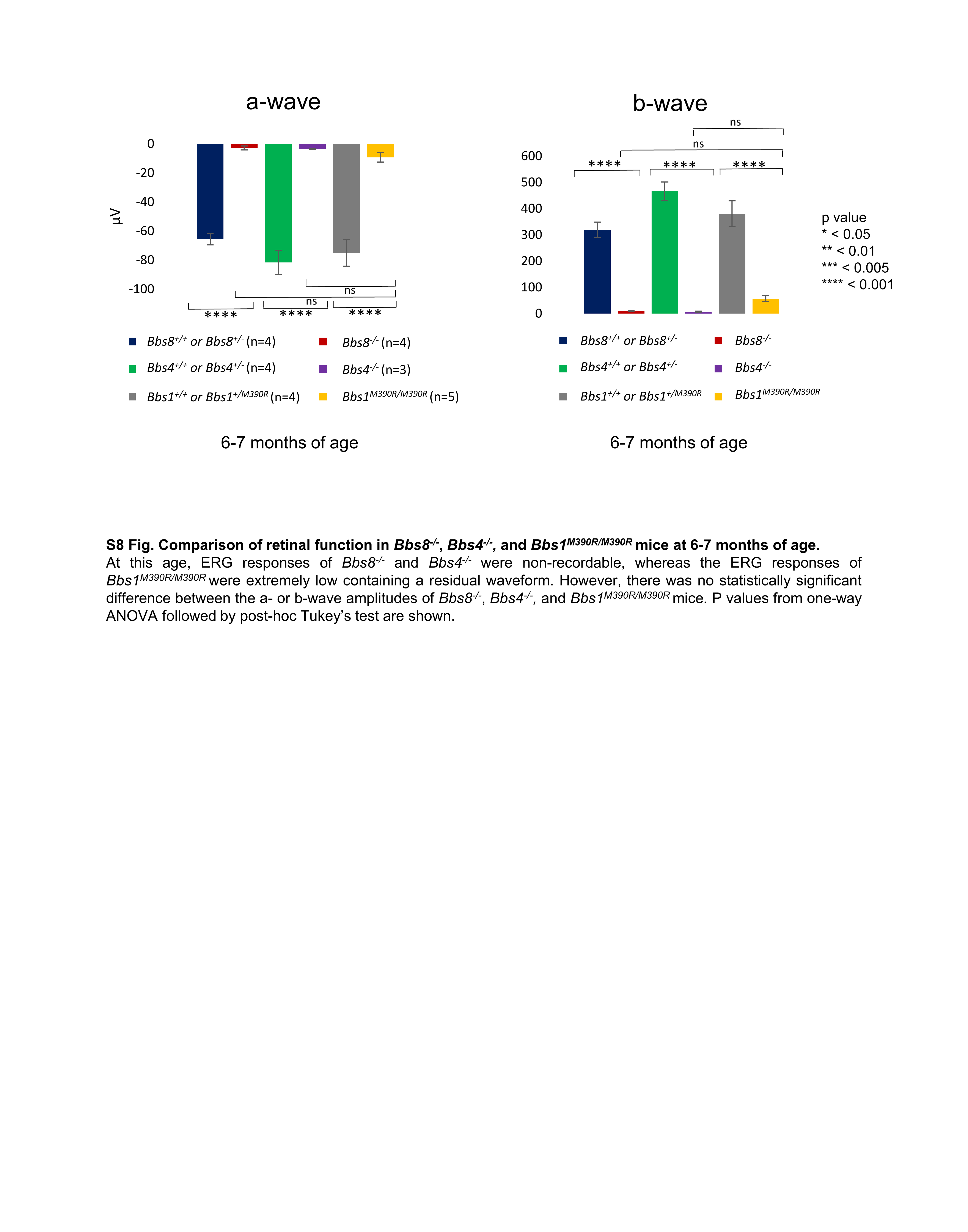

Supplement: S8 Fig — At this age, ERG responses of Bbs8-/- and Bbs4-/- were non-recordable, whereas the ERG responses of Bbs1M390R/M390R were extremely low containing a residual waveform. However, there was no statistically significant difference between the a- or b-wave amplitudes of Bbs8-/-, Bbs4-/-, and Bbs1M390R/M390R mice. P values from one-way ANOVA followed by post-hoc Tukey’s test are shown. (TIF) [file pgen.1007057.s008.TIF]

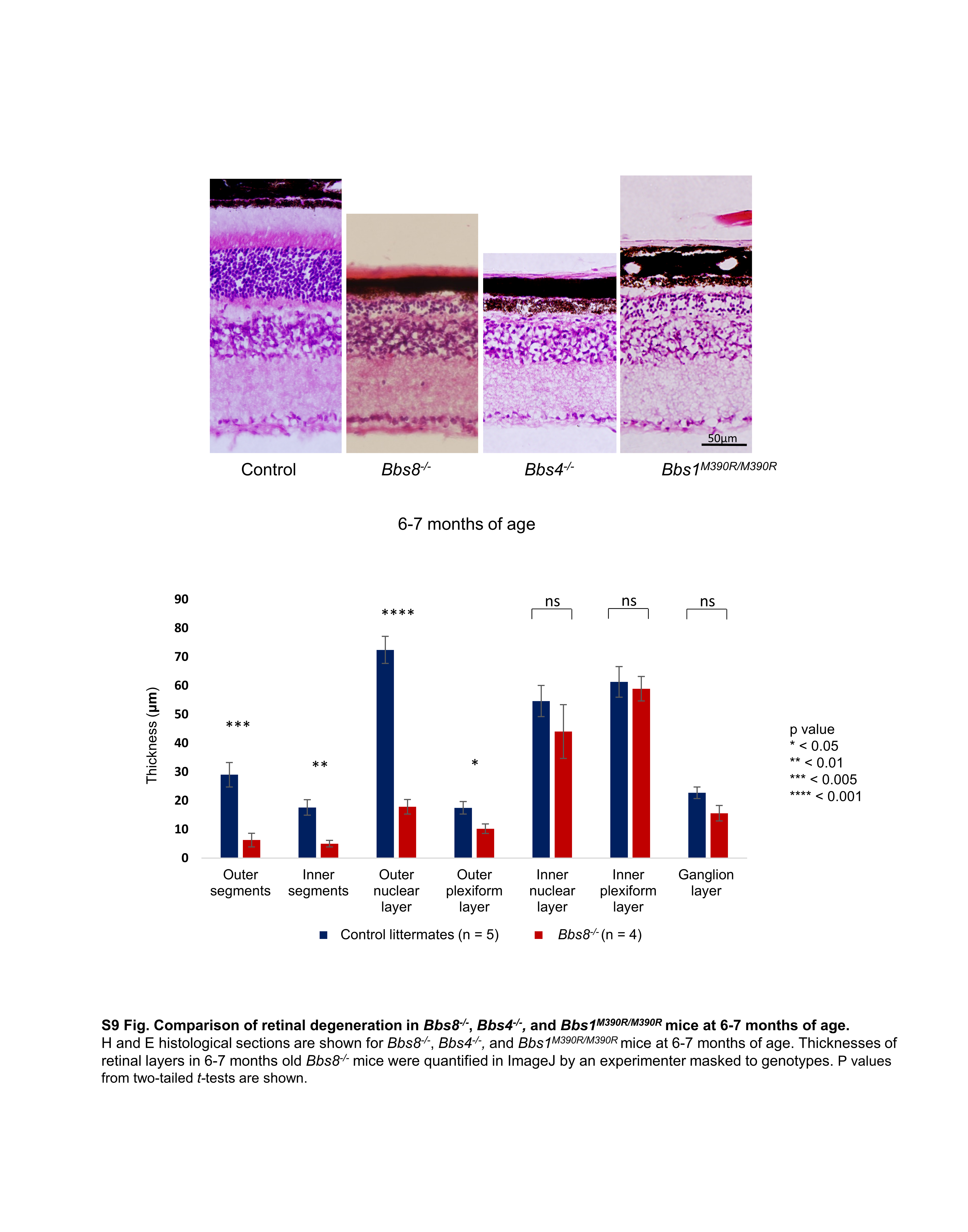

Supplement: S9 Fig — H and E histological sections are shown for Bbs8-/-, Bbs4-/-, and Bbs1M390R/M390R mice at 6–7 months of age. Thicknesses of retinal layers in 6–7 months old Bbs8-/- mice were quantified in ImageJ by an experimenter masked to genotypes. P values from two-tailed t-tests are shown. (TIF) [file pgen.1007057.s009.TIF]

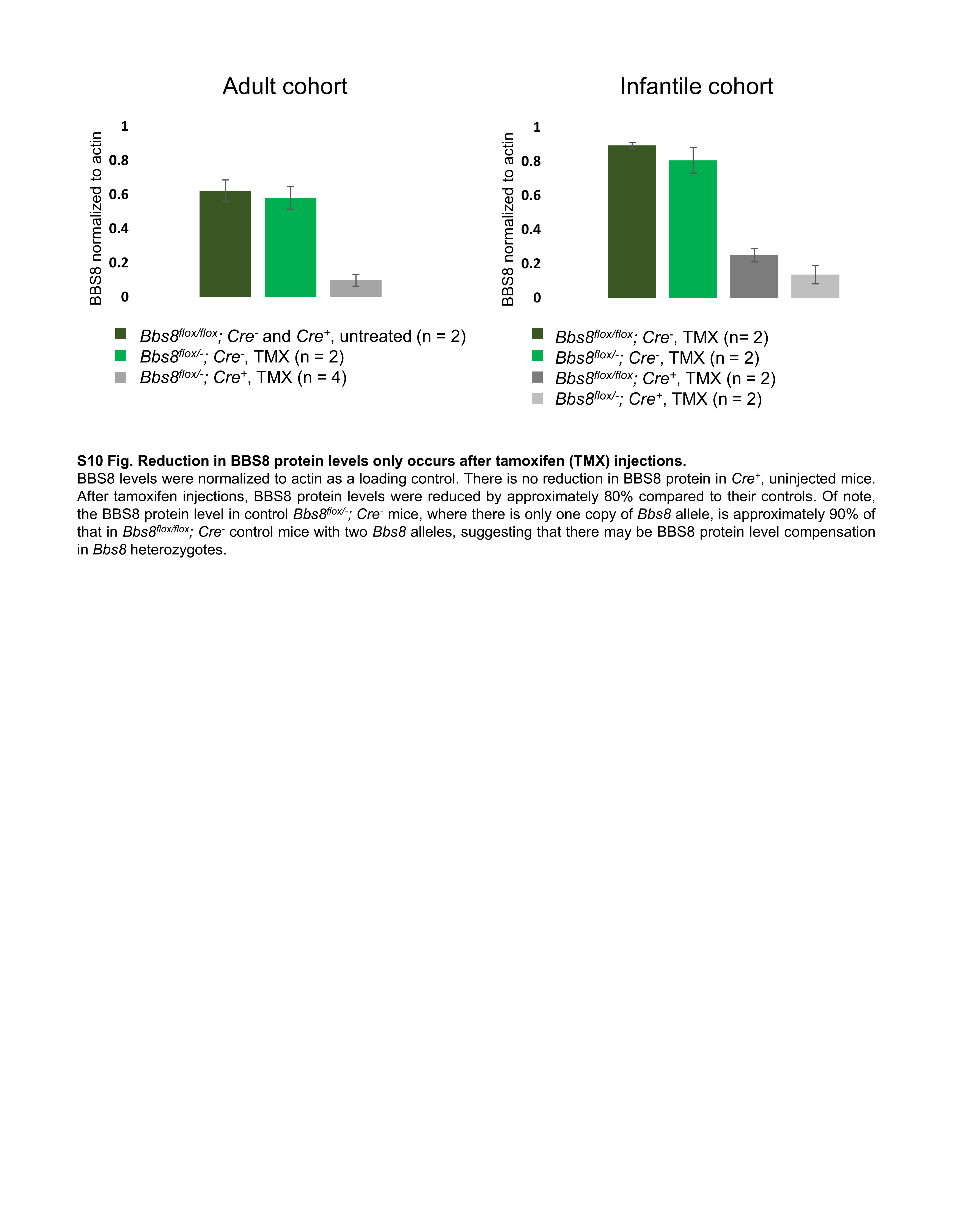

Supplement: S10 Fig — BBS8 levels were normalized to actin as a loading control. There is no reduction in BBS8 protein in Cre+, uninjected mice. After tamoxifen injections, BBS8 protein levels were reduced by approximately 80% compared to their controls. Of note, the BBS8 protein level in control Bbs8flox/-; Cre- mice, where there is only one copy of Bbs8 allele, is approximately 90% of that in Bbs8flox/flox; Cre- control mice with two Bbs8 alleles, suggesting that there may be BBS8 protein level compensation in Bbs8 heterozygotes. (TIF) [file pgen.1007057.s010.TIF]

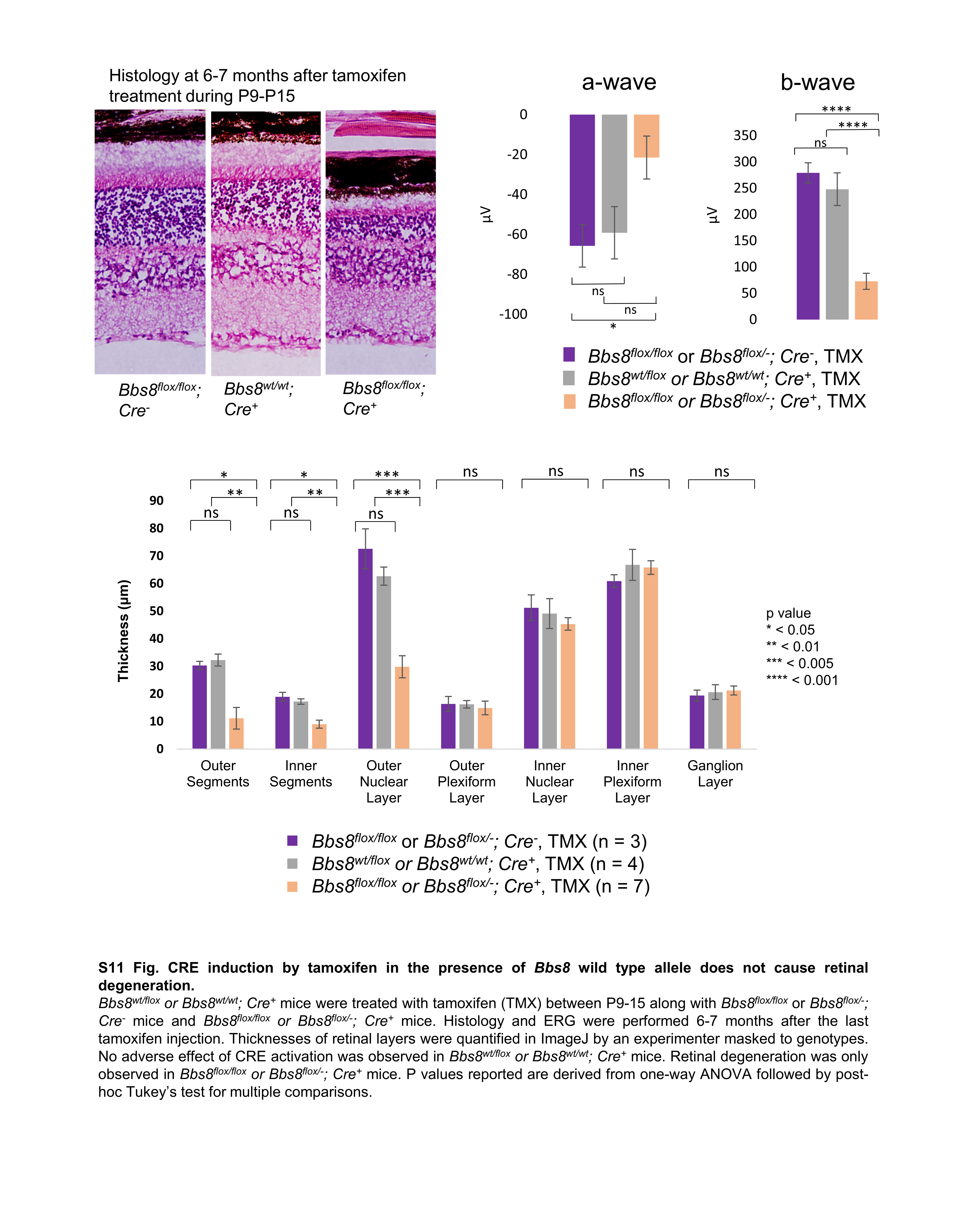

Supplement: S11 Fig — Bbs8wt/flox or Bbs8wt/wt; Cre+ mice were treated with tamoxifen (TMX) between P9-15 along with Bbs8flox/flox or Bbs8flox/-; Cre- mice and Bbs8flox/flox or Bbs8flox/-; Cre+ mice. Histology and ERG were performed 6–7 months after the last tamoxifen injection. Thicknesses of retinal layers were quantified in ImageJ by an experimenter masked to genotypes. No adverse effect of CRE activation was observed in Bbs8wt/flox or Bbs8wt/wt; Cre+ mice. Retinal degeneration was only observed in Bbs8flox/flox or Bbs8flox/-; Cre+ mice. P values reported are derived from one-way ANOVA followed by post-hoc Tukey’s test for multiple comparisons. (TIF) [file pgen.1007057.s011.tif]

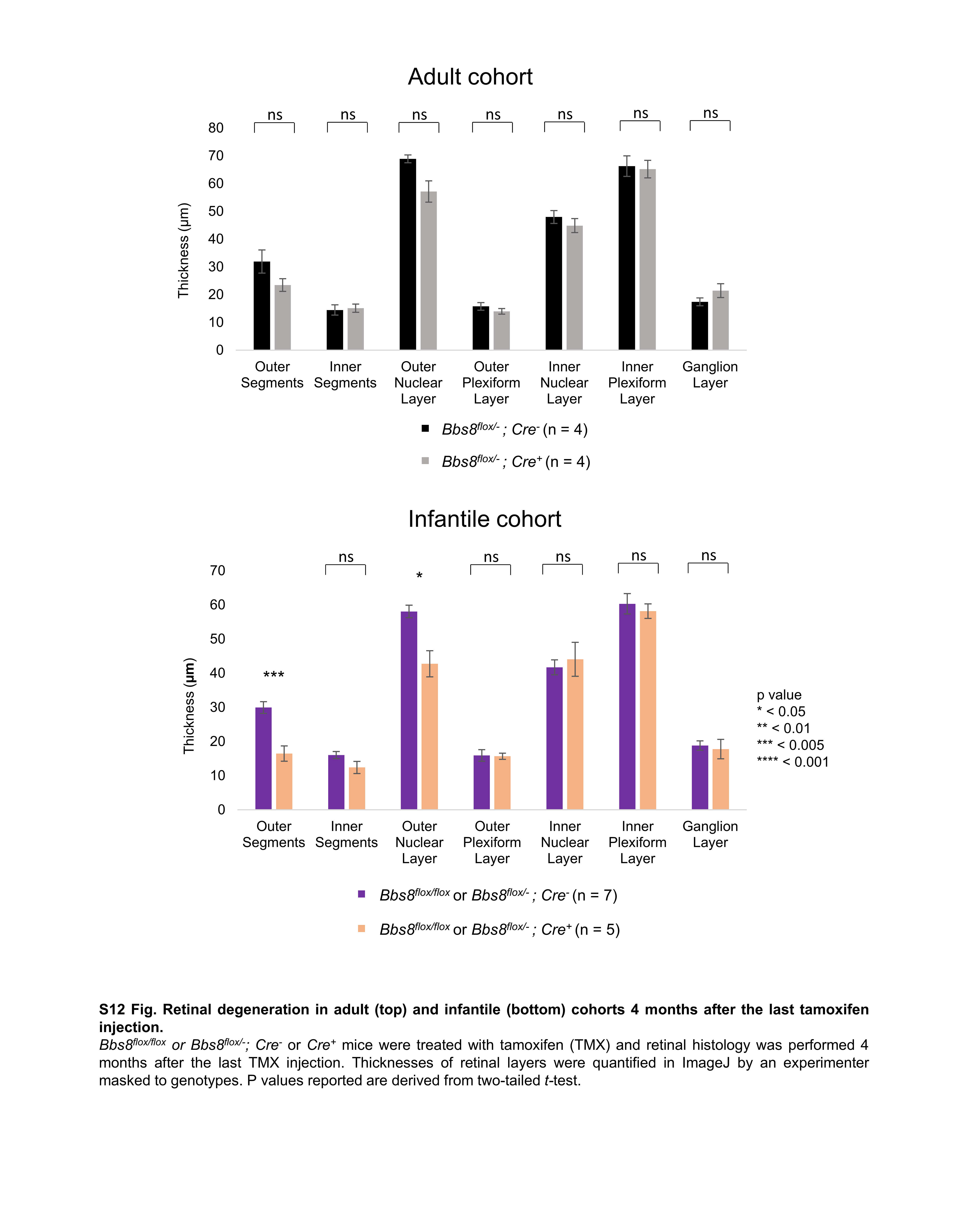

Supplement: S12 Fig — Bbs8flox/flox or Bbs8flox/-; Cre- or Cre+ mice were treated with tamoxifen (TMX) and retinal histology was performed 4 months after the last TMX injection. Thicknesses of retinal layers were quantified in ImageJ by an experimenter masked to genotypes. P values reported are derived from two-tailed t-test. (TIF) [file pgen.1007057.s012.tif]

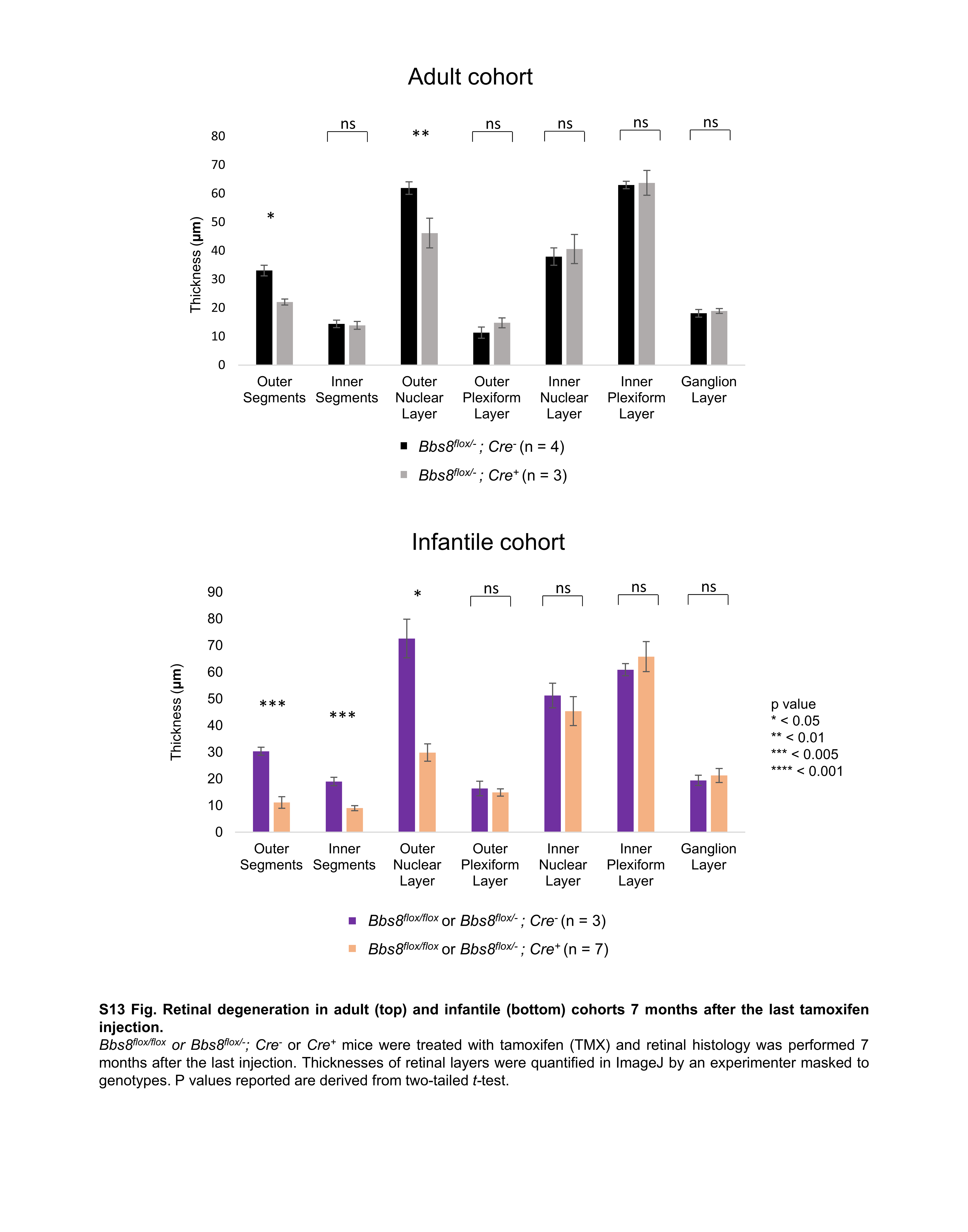

Supplement: S13 Fig — Bbs8flox/flox or Bbs8flox/-; Cre- or Cre+ mice were treated with tamoxifen (TMX) and retinal histology was performed 7 months after the last injection. Thicknesses of retinal layers were quantified in ImageJ by an experimenter masked to genotypes. P values reported are derived from two-tailed t-test. (TIF) [file pgen.1007057.s013.TIF]

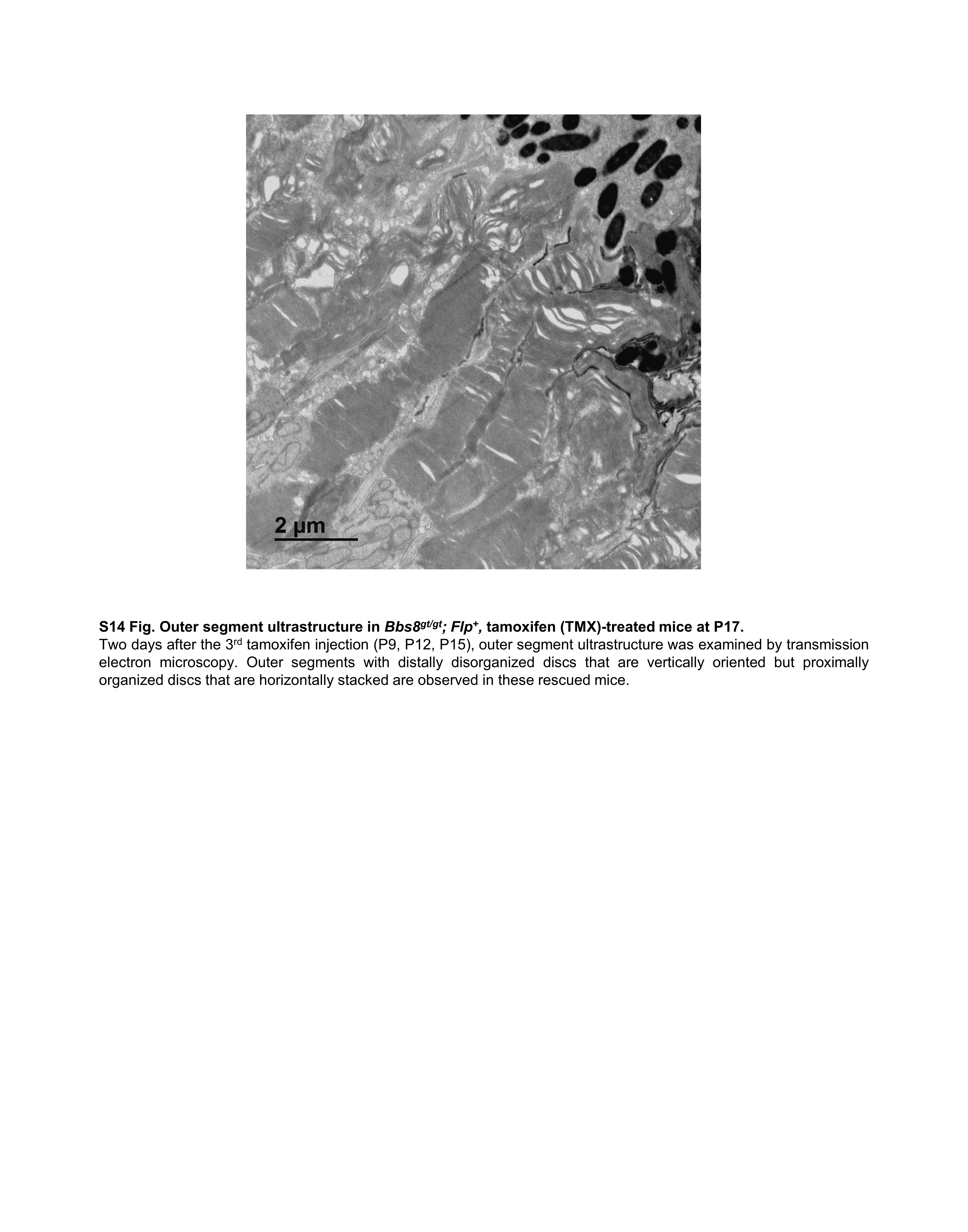

Supplement: S14 Fig — Two days after the 3rd tamoxifen injection (P9, P12, P15), outer segment ultrastructure was examined by transmission electron microscopy. Outer segments with distally disorganized discs that are vertically oriented, but proximally organized discs that are horizontally stacked are observed in these rescued mice. (TIF) [file pgen.1007057.s014.TIF]
